# Scale effect of circularly polarized luminescent signal of matter

**DOI:** 10.1093/nsr/nwad072

**Published:** 2023-03-17

**Authors:** Siyu Sun, Xiaolin Li, Chen Xu, Yan Li, YongZhen Wu, Ben L Feringa, He Tian, Xiang Ma

**Affiliations:** Key Laboratory for Advanced Materials and Feringa Nobel Prize Scientist Joint Research Center, Frontiers Science Center for Materiobiology and Dynamic Chemistry, School of Chemistry and Molecular Engineering, East China University of Science and Technology, Shanghai 200237, China; School of Physics, East China University of Science and Technology, Shanghai 200237, China; Key Laboratory for Advanced Materials and Feringa Nobel Prize Scientist Joint Research Center, Frontiers Science Center for Materiobiology and Dynamic Chemistry, School of Chemistry and Molecular Engineering, East China University of Science and Technology, Shanghai 200237, China; Key Laboratory for Advanced Materials and Feringa Nobel Prize Scientist Joint Research Center, Frontiers Science Center for Materiobiology and Dynamic Chemistry, School of Chemistry and Molecular Engineering, East China University of Science and Technology, Shanghai 200237, China; Key Laboratory for Advanced Materials and Feringa Nobel Prize Scientist Joint Research Center, Frontiers Science Center for Materiobiology and Dynamic Chemistry, School of Chemistry and Molecular Engineering, East China University of Science and Technology, Shanghai 200237, China; Key Laboratory for Advanced Materials and Feringa Nobel Prize Scientist Joint Research Center, Frontiers Science Center for Materiobiology and Dynamic Chemistry, School of Chemistry and Molecular Engineering, East China University of Science and Technology, Shanghai 200237, China; Stratingh Institute for Chemistry and Zernike Institute for Advanced Materials, Faculty of Science and Engineering, University of Groningen, Groningen, AG 9747, Netherlands; Key Laboratory for Advanced Materials and Feringa Nobel Prize Scientist Joint Research Center, Frontiers Science Center for Materiobiology and Dynamic Chemistry, School of Chemistry and Molecular Engineering, East China University of Science and Technology, Shanghai 200237, China; Key Laboratory for Advanced Materials and Feringa Nobel Prize Scientist Joint Research Center, Frontiers Science Center for Materiobiology and Dynamic Chemistry, School of Chemistry and Molecular Engineering, East China University of Science and Technology, Shanghai 200237, China

**Keywords:** scale effect, circularly polarized luminescent, chirality, quantum dots, perovskite

## Abstract

Circularly polarized luminescence (CPL) is an important part in the research of modern luminescent materials and photoelectric devices. Usually, chiral molecules or chiral structures are the key factors to induce CPL spontaneous emission. In this study, a scale-effect model based on scalar theory was proposed to better understand the CPL signal of luminescent materials. Besides chiral structures being able to induce CPL, achiral ordered structures can also have a significant influence on CPL signals. These achiral structures are mainly reflected in the particle scale in micro-order or macro-order, i.e. the CPL signal measured under most conditions depends on the scale of the ordered medium, and does not reflect the inherent chirality of the excited state of the luminescent molecule. This kind of influence is difficult to be eliminated by simple and universal strategies in macro-measurement. At the same time, it is found that the measurement entropy of CPL detection may be the key factor to determine the isotropy and anisotropy of the CPL signal. This discovery would bring new opportunities to the research of chiral luminescent materials. This strategy can also greatly reduce the development difficulty of CPL materials and show high application potential in biomedical, photoelectric information and other fields.

## Teaser

A scale-effect model based on scalar theory can help understand the CPL signal, and measurement entropy of CPL detection may determine the isotropy and anisotropy of the CPL signal.

## INTRODUCTION

**Figure 1.**
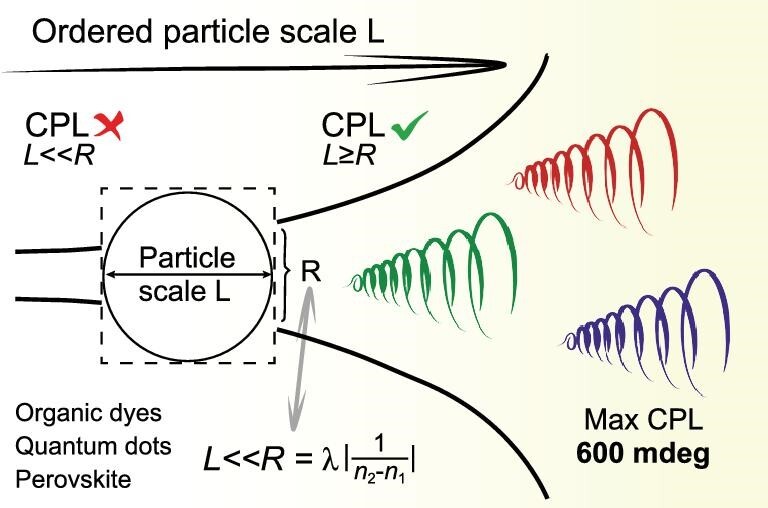
Schematic diagram of the scale-effect model based on scalar theory.

The asymmetry of matter has gradually attracted the attention of a wide range of researchers in materials science and chemistry [1]. Recently, many important applications have been reported that take advantage of chiral materials and circularly polarized luminescence/light (CPL) [2,3] and the CPL emission of materials has also seen broad interest [1,4–13]. For the CPL emission of materials, there is a consensus that the observed CPL signal is mainly due to the intrinsic asymmetric nature of molecules [14] or the asymmetry induced by the ordered supramolecular assembly [5,15–18]; in addition, there is also the view that a supramolecular assembly based on small chiral molecules can effectively amplify or transfer the CPL signal of these materials [19]. However, in this paper, we show that the CPL signal of many luminescent materials is affected by the dielectric properties and anisotropy scale of the materials (Fig. 1) but not just from the molecular chirality induced. The CPL signal of the anisotropic structure formed by aggregation or assembly of luminescent materials is constrained by the critical anisotropy scale (*R*). When the anisotropy scale (*L*) of material particles is greater than or equal to this critical scale, the anisotropic environment has a great influence on the CPL signal. Only when $L \ll R$ is the influence of the anisotropic environment on the CPL signal negligible. For nonmagnetic luminescent materials with no absorption at a specific wavelength, the critical scale *R* is affected by the dielectric function of the anisotropic environment of the luminescent materials. The dielectric distribution of luminescent materials depends on the molecular structure and the ordering in the assembly. In addition, we propose a method of artificially introducing anisotropy, such as by introducing a magnetic field and changing the dielectric function distribution—a highly universal strategy for developing CPL spontaneous radiation materials with controllable CPL emission wavelength and signal intensity.

The understanding of CPL by different investigators varies considerably [20,21]. A mechanical interpretation of CPL luminescence produced under different conditions is complicated, has low universality and lacks relevance between the mechanisms in different systems. At present, many studies only consider the contribution of molecular intrinsic chirality or chiral assembly to the CPL signal, but ignore the contribution of achiral structure to the CPL signal. Besides, the development of CPL materials is largely based on trial and error, and it is still difficult to develop customized CPL materials that can be utilized in practical applications. The research presented here provides a new perspective on the treatment and development of CPL luminescent materials and provides a reliable and uniform explanation for many of the difficult-to-explain phenomena in current CPL material research. It also provides a theoretical explanation based on the scale-dependent effect for the phenomenon that the chiral CPL signal is generated by the chiral assembly of achiral materials (Section 5 in Supporting information). Based on this approach, a universal strategy for the preparation of customized CPL materials by inducing environmental anisotropy is proposed. This discovery will promote the development of aggregated state chemistry [22] and help researchers to focus on the scale effect of an anisotropic environment of CPL luminescent materials to display the wider application prospects for many fields ranging from optoelectronic and luminescent devices to biomedical materials.

## THEORETICAL ANALYSIS

### Scale-effect model

First, the influence of the anisotropic environment (including microscopic-ordered assembly or aggregation, etc.) on the polarization properties (including CPL parameters of the CPL signal) of electromagnetic waves is not negligible. Therefore, a quantifiable model and an explanation based on previous research [23] and a field theory derivation are presented, and the model together with the inference based on this model are subsequently supported by experimental data.

To quantitatively determine the influence of the particle scale in uniform anisotropic media on CPL spontaneous radiation, Equation (1) was obtained in nonmagnetic media and transparent media. Please refer to the supporting information for the derivation process of Equation (1):


(1) \begin{eqnarray*} L \ll R\ = {\rm{\ }}\lambda \left| {\frac{1}{{{n}_2 - {n}_1}}} \right|, \end{eqnarray*}

where ${n}_1$ and ${n}_2$ are the two intrinsic refractive indices for orthogonally polarized electromagnetic waves $\overrightarrow {{D}_1} $ and $\overrightarrow {{D}_2} $ generated in the $\hat{n}$ propagation direction in the anisotropic medium, $\lambda $ is the wavelength of electromagnetic waves transmitted in the medium, *L* is the transmission distance in the medium (anisotropy scale) and *R* is the critical scale of the anisotropic environment.

When $L <\! < R$, the effect of the resulting homogeneous anisotropic medium on the finally detected CPL signal can be neglected. Otherwise, the effect of the anisotropic medium on the CPL signal detected in the $\hat{n}$ direction is large and non-negligible. After the electromagnetic waves leave the anisotropic medium and enter a vacuum or an isotropic medium, in which ${n}_1 \approx {n}_2$, the two waves finally merge into one elliptically polarized wave. Therefore, the phase difference between the two waves in the anisotropic medium will affect the elliptically polarized wave detected by the instrument. For the detailed discussion about the scale-effect model based on scalar theory of CPL emission, please see supporting information, and a specific case based on the data reported in the literature is also given in the supporting information.

Combined with the above considerations, some qualitative conclusions can be obtained about the influence of the anisotropic environment on the CPL signal of luminescent materials in the visible band and near-infrared band:

- When the anisotropic environment has a definite influence on the phase of polarized light arising from ${n}_1\tilde {n}_2$, the influence of the anisotropic environment on the phase is not affected by the wavelength of the electromagnetic waves.
- Regarding the influence of the polarity ($\varepsilon $) of anisotropic media on CPL signals at the same scale, the influence of a low-polarity anisotropic environment on CPL signals is much greater than that of a high-polarity anisotropic environment.

### In chiral assembly

The scale-effect model based on scalar theory also provides an efficient explanation for the phenomena of the chiral assembly-induced CPL emission of the achiral materials. These phenomena in the scale-effect model could be considered as the formation of the media with chiral dielectric tensor distribution. The media, as the mirror images of chiral helical assembly structures [24], could induce CPL signals with opposite signs and equal strength can also be proven by using the scale-effect model of CPL emission (see Section 5 in the Supporting information for the derivation process). Taking a chiral helical assembly structure as an example, the phase difference of polarized light due to the environment can be easily proven to have the same modulus and opposite sign in media with reciprocal chiral dielectric tensor distributions. The properties of the dielectric tensor of CPL luminescent materials with enantiomorphic dielectric distributions are reflected in the actual experiment as CPL signals with opposite signs. Induction of CPL emission from organic dyes without chiral structures in a microcrystalline state has been reported to be feasible by using a chiral macrocyclic environment [25] or hierarchical structure of polycrystals [7].

### Measurement entropy

As it is well known that linear polarized light (LPL) and CPL are the special elliptically polarized light (EPL) with a special phase difference between electric vector $\overrightarrow {{D}_1} $ and $\overrightarrow {{D}_2} $. Our research, based on the scale effect of the spontaneous radiation of material, established that the corresponding CPL or LPL signal could be measured when the scale of the medium meets the value shown in Supplementary Equation (S2). With the increase in the scale of the medium, the corresponding produced CPL (or LPL) could be converted into LPL (or CPL). First, the simplest case was considered here. When the resulting phase difference ${\rm{\Delta }}\varphi \in ( {0,{\rm{\pi }}} ]$, which is $n\ = {\rm{\ }}1$, Supplementary Equations (S3) and (S4) are the minimum medium scales to generate CPL and LPL. It is easy to observe that ${L}_{CPL} < {L}_{LPL}$ could be obtained with Supplementary Equation (S4) minus Supplementary Equation (S3). It can be found that CPL luminescence is generated first compared with LPL as the scale of the medium increases under conditions that do not consider EPL. Then, in the condition of ${\rm{\Delta }}\varphi \in ( {0,{\rm{\pi }}} ]$ and $L < {L}_{LPL}$, the spontaneous radiation produced in the medium can only be EPL and CPL. When under the condition of $L > {L}_{LPL}$, the spontaneous radiation produced from the medium was the superposition of LPL, CPL and general EPL (${\rm{\Delta }}\varphi \ne \frac{{{\rm{n\pi }}}}{2}$). Therefore, it is significant to discuss the occupancy ratio from CPL, LPL to general EPL. The ratio function${\rm{\ \xi }}$ (Supplementary Equation (S6)) of CPL and LPL relative to EPL could be obtained according to the differential of the period of phase difference such as in Supplementary Equation (S5). Supplementary Equation (S7) is the ratio function ${\rm{\xi }}$ of CPL and LPL relative to EPL. It is easy to recognize that the polarization signals we measured (vide infra) were mainly general EPL, whereas the contribution of CPL and LPL was essentially none. The explanation that EPL is predominant could be used to understand the differences between the anisotropic and isotropic signals in the measurement of polarized light that radiated from spontaneous radiation materials. The measurement entropy of the materials could be the correct interpretation of this observation. (For a detailed discussion of measurement entropy, see the supporting information.) For the superposition state of many EPL signals, they represent an anisotropic character depending on the long axis and short axis of the corresponding ellipse. The isotropic character could represent the condition when the measurement entropy is large (Supplementary Fig. S14). Corresponding literature also reported that the anisotropic and isotropic polarized signals were observed under the condition of small measurement entropy and large measurement entropy, respectively [26].

The influence of the scale effect of the medium on the spontaneous emission of the material needs to consider not only the influence of the anisotropic distribution of the medium on the polarization state of the spontaneous emission, but also the polarization state of the incident excitation light (see supporting information).

## RESULTS AND DISCUSSION

### In periodic structure

**Figure 2.**
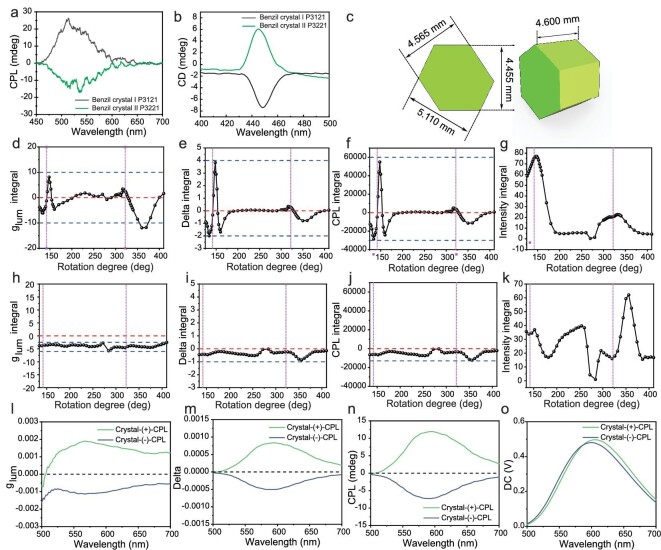
Tunable CPL signal of photoluminescent materials in a periodic structure. (a) CPL spectra and (b) CD spectra of benzil crystals with different space point groups (P3121 and P3221). (c) 3D model and detailed measurement data of the model benzil crystal (a screw micrometer was used for measurement, and the measurement error was <0.01 mm). The optical axis of the crystal is perpendicular to the hexagonal plane of the crystal according to the angle-dependent CPL spectra study. (d) Integral glum value, (e) integral delta, (f) integral CPL signal and (g) integral light intensity from the angle-dependent CPL spectra study in which the angle between the incident light and the optical axis of the model crystal was changed, wherein the *x*-axis is the scale on a 2D platform (Supplementary Fig. S3; the measurement error is <1 deg) and 142° is the normal incident angle on the scale parallel to the optical axis (for the detailed CPL spectra, please see Supplementary Figs S6–S9). (h) Integral glum value, (i) integral delta, (j) integral CPL signal and (k) integral light intensity from the angle-dependent CPL spectra study in which the angle between the incident light and optical axis of the model crystal was fixed at 90° (for the detailed CPL spectra, please see Supplementary Figs S10–S13). (l) Glum spectra, (m) delta spectra, (n) CPL spectra and (o) light-intensity spectra of light transmitted through the model benzil crystal (*λ*_transmitted_ = 530 nm).

CPL signals with opposite signs can be observed by preparing crystals from achiral benzil with different space point groups (P3_1_21 and P3_2_21) when using the same crystal plane as the incidence direction (Fig. 2a and b). This is consistent with the phenomenon of existing studies that luminescent molecules produce corresponding CPL in enantiomeric periodic structures, which could be due to the birefringence phenomenon of the crystal. In addition, spontaneous CPL of a single benzil crystal was verified in experiments. The size of the benzil crystals tested in the experiments is above the order of magnitude of ${10}^{ - 3}$ meters, and the periodic anisotropic structure of this size cannot be ignored for the spontaneous radiation from benzil crystals under the dielectric distribution of low-polarity organic molecules. Generally, a CPL spectrometer detects the polarization state of electromagnetic waves along a fixed optical path, so it can be assumed that the polarization state of electromagnetic waves detected by the CPL spectrometer is $\hat{n}$. A benzil crystal is a uniaxial crystal, which means that the propagation properties of all electromagnetic waves in benzil are subject to the propagation characteristics of a uniaxial crystal. When the angle between $\hat{n}$ and the optical axis of the crystal changes, ${n}_1$ and ${n}_2$ will change. This can be understood as the propagation speeds of electromagnetic waves $\overrightarrow {{E}_1} $ and $\overrightarrow {{E}_2} $ of the same frequency in the same propagation direction being different, so the generated phase difference changes. This is proven by the angle-dependent CPL spectroscopy observation of reversal of the CPL signal with changes in the angle (Fig. 2d–k). Due to the characteristics of uniaxial crystal symmetry, ${n}_1 = {n}_o{\rm{\ }} = \sqrt {{{\rm{\varepsilon }}}_ \bot } \ $, and ${n}_2 = {n}_e $, where the size of ${n}_e$ depends on the angle $\theta $ between $\hat{n}$ and the optical axis [27]:


(2) \begin{eqnarray*} {\rm{\ }}\frac{1}{{{n}_e^2}} = \frac{{{{\sin }}^2{\rm{\theta }}}}{{{\varepsilon }_\parallel }}{\rm{\ }} + \frac{{{{\cos }}^2{\rm{\theta }}}}{{{\varepsilon }_ \bot }}. \end{eqnarray*}

Therefore, when the angle between the optical axis of the crystal and the incident angle changes, the CPL signal reverses positively and negatively (Fig. 2h–k). In addition, the transmitted natural light incident on the same crystal at different angles indicates that the transmitted light will also produce polarization signal changes under various environmental effects of the periodic structure (Fig. 2l–o), verifying that the anisotropy of the periodic structure has an important impact on CPL spontaneous radiation. Furthermore, the lifetime of spontaneous radiation, orientation of the molecular transition dipole, defects of the anisotropic structure and effect of Snell's law under the interface condition all affect the CPL signal of the system.

### In aggregate state

Next, the effect of anisotropic aggregates dispersed in an isotropic medium on the CPL signal of the system was analysed. Benzil has high solubility in dichloromethane, a low-polarity organic solvent, and no CPL signal was detected in the dichloromethane solution of benzil with a concentration of 10^−2^ M. Benzil molecules with the same crystal form were uniformly dissolved in ethanol solution and rapidly doped in a polyvinyl alcohol (PVA) film; CPL signals corresponding to the crystal structure could be measured from the film (Supplementary Fig. S15). Due to the low solubility of benzil molecules in a highly polar solvent, they spontaneously aggregate in the solution and form anisotropic aggregates with large sizes ($L \ge R$) in the doping process.

The control experiment proves that the untreated PVA film is an isotropic medium in the visible light band and does not affect the polarization signal of the CPL material doped in it. The CPL signal produced by the benzil@PVA film can be understood to be influenced by the larger anisotropic aggregates in the isotropic solid medium.

### Universal strategy for CPL materials

Based on Equation (1), we developed a highly universal strategy for developing CPL spontaneous radiation materials with controllable CPL emission wavelengths and controllable signals, i.e. artificially introducing anisotropy to influence CPL signals. In this strategy, molecules or aggregate-state luminescent materials ($L{\rm{\ }} \ll {\rm{\ }}R$) are doped into an isotropic medium (such as an amorphous matrix or a solution) and the spontaneous radiation light detected by the CPL spectrometer corresponds to $L\ = {L}_{{\rm{aggregates}}}{\rm{\ }} \ll R$, so no CPL signal is detected. If artificial anisotropy is introduced into the isotropic medium, taking a spatial anisotropy distribution as an example, then the dielectric tensor of the artificially anisotropic medium will become a second-order tensor function of position $\varepsilon ( {\omega ,\vec{r}} )$, in contrast to the constant dielectric tensor $\varepsilon ( \omega )$ in the uniform isotropic medium. For spontaneous emission of light in the $\hat{n}$ direction, *L* changes and $L\ = {L}_{{\rm{aggregate}}}{\rm{\ }} + {L}_{{\rm{medium}}}$. Methods of introducing anisotropy, such as introducing a magnetic field, changing the dielectric function distribution [25,28] and other means, as well as the size of the isotropic medium, will affect the CPL signal. For instance, it has been reported that the CPL signals were detected from ferroelectric polarization-induced anisotropy in ferroelectric-based perovskite materials [29]. We chose the thin-film stretching method to introduce macroscopic anisotropy, where $\frac{d}{{d\vec{r}}}[ {\varepsilon ( {\omega ,\vec{r}} )} ] \ne 0$ and ${L}_{{\rm{medium}}} \gg {L}_{{\rm{aggregate}}}$, and the anisotropic environment of the medium plays a major role in the CPL signal. In addition, the influence of macro-anisotropy on the CPL signal is discussed for microscopic dispersion and microscopic aggregation states by selecting materials with different luminescence mechanisms.

**Figure 3.**
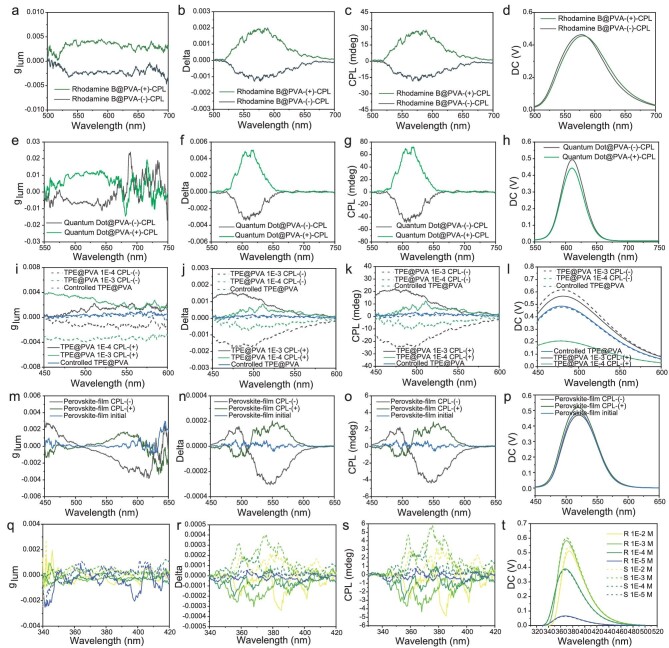
Tunable CPL signal of luminescent materials induced by an anisotropic environment. (a) Glum spectra, (b) delta spectra, (c) CPL spectra and (d) light-intensity spectra of the film doped with RhB without a chiral structure into a PVA matrix and in which an anisotropic environment is produced by stretching. (e) Glum spectra, (f) delta spectra, (g) CPL spectra and (h) light-intensity spectra of quantum dots (*λ*_em_ = 610 nm) in a PVA matrix after an anisotropic environment is generated by stretching. (i) Glum spectra, (j) delta spectra, (k) CPL spectra and (l) light-intensity spectra of TPE@PVA films with different doping molar concentrations. (m) Glum spectra, (n) delta spectra, (o) CPL spectra and (p) light-intensity spectra of perovskite-based luminescent films (*λ*_ex_ = 365 nm, *λ*_em_ = 520 nm) when an anisotropic transparent PVA film was added into the optical path between the detector and the luminescent sample. (q) Glum spectra, (r) delta spectra, (s) CPL spectra and (t) light-intensity spectra of (S/R)-2,2'-diethoxy-1,1'-binaphthyl dissolved in a dichloromethane solution at different concentrations.

Here, the amorphous organic film PVA is taken as an example. Previous experiments have proven that an untreated PVA film does not affect the CPL signal in the visible light band. Rhodamine B (RhB), CdSe quantum dots, aggregation-induced emission (AIE) tetraphenyl ethylene (TPE) dyes and perovskite-based luminescent films with different luminescent mechanisms were elected here to prove this strategy. Figure 3a–p is the CPL spectra which prove that the luminescent materials with different emission mechanisms all have opposite CPL signals (see supporting information). This provides a different argument for the introduction of the macro-anisotropy scale to develop CPL emission. In addition, Fig. 3q–t is the concentration-dependent CPL spectroscopy study of a pair of chiral molecules, proving that the scale-effect model could also be another simple and clear choice to explain the CPL emission in solvents (see supporting information).

## CONCLUSION AND PROSPECTIVE

In previous studies, the origin of CPL emission and the chirality of materials were usually discussed as related phenomena. Besides the chiral-induced CPL emission phenomena, we found that the scale effect based on the anisotropy of the medium is found to be another key factor to induce a CPL signal. Additionally, it is easy to conclude that a pair of chiral structures can induce CPL emission using an analysis based on the scale-effect model emanating from scalar theory. Therefore, these findings can also explain the reason why a pair of chiral structures can induce CPL signals with opposite signs and equivalent intensity from another angle. In addition, we found that the measured entropy of materials is the key factor that determines the isotropy or anisotropy of measured CPL signals, rather than the influence of LPL. It is shown here that the influence of the anisotropic environment on the CPL signal is extremely important in the development of CPL luminescent materials. When luminescent materials orderly aggregate, an anisotropic medium environment will be formed. The anisotropic medium environment will change the refractive indices ${n}_1$ and ${n}_2$ of two orthogonally polarized electromagnetic waves propagating in the same direction. In other words, the propagation speeds of the two electromagnetic waves in the medium are different, so when the propagation distance is relatively long, the different phase differences along the optical path will affect the CPL signal of the material. The ${n}_1$ and ${n}_2$ of the dielectric material and the electromagnetic wave wavelength $\lambda $ jointly determine the influence of the anisotropic environment on the CPL model. When the constraint of $L{\rm{\ }} \ll {\rm{\ }}R$ is met, the impact of the anisotropic environment on the CPL signal can be ignored. It is also discovered that the CPL signal measured from the CPL spectra is essentially not the signal of circularly polarized light, but is EPL in the vast majority of cases. Furthermore, the CPL signal of the EPL is highly dependent on the phase difference and amplitude of the orthogonal component of the electric field. The measurement entropy of the CPL signal is the main factor to determine whether the polarized state of the radiation is anisotropic or isotropic. When the measurement entropy is small, the polarized signal is isotropic; and when the measurement entropy is large, the polarized signal is anisotropic.

We selected luminescent materials with different luminescence mechanisms, including but not limited to organic dyes that emit fluorescence and phosphorescence light as well as inorganic luminescent materials, for verification of our theory. In addition, we proposed a method of artificially introducing anisotropy, such as introducing a magnetic field and changing the dielectric function distribution, and developed a highly universal strategy for developing CPL spontaneous radiation materials with controllable CPL emission wavelength and signal.

This discovery will further expand the application range of CPL luminescent materials. For example, the CPL signal of different wavelengths of dyes in an anisotropic environment can be used to observe the dielectric distribution and anisotropic scale of a microscopic-ordered structure. Furthermore, different cells with different anisotropic scales can be stained and the CPL signals of spontaneous radiation can be observed to quickly determine the size of different cells.

## MATERIALS AND METHODS

Detailed materials and methods are available in the Supplementary data (2022-11-16 CPL SI.docx). The single-crystal data are available in Benzil P3121.cif and Benzil P3221.cif.

## Supplementary Material

nwad072_Supplemental_Files
