# Supplementary material for: Scale effect of circularly polarized luminescent signal of matter
Source: Natl Sci Rev. 2023 Mar 17;10(5):nwad072. doi: 10.1093/nsr/nwad072 (PMC10243995; doi:10.1093/nsr/nwad072)
Supplement: nwad072_Supplemental_Files [file nwad072_supplemental_files.zip › 2022-11-16 CPL SI.docx]

**Supporting Information**

**Scale-effect of Circular Polarized Luminescent Signal of Matter**

Siyu Sun^1+^, Xiaolin Li^2+^, Chen Xu^1^, Yan Li^1^, YongZhen Wu^1^, Ben L. Feringa^1,3^, He Tian^1^, Xiang Ma*^1^

^1^Key Laboratory for Advanced Materials and Feringa Nobel Prize Scientist Joint Research Center, Frontiers Science Center for Materiobiology and Dynamic Chemistry, School of Chemistry and Molecular Engineering, East China University of Science and Technology, Meilong Road 130, Shanghai, 200237 China

^2^School of Physics, East China University of Science and Technology, Meilong Road 130, Shanghai, 200237 China

^3^Stratingh Institute for Chemistry and Zernike Institute for Advanced Materials, Faculty of Science and Engineering, University of Groningen, Nijenborgh 4, Groningen, AG, 9747 Netherlands

**Contents**

[1. Experimental Section 2](#_Toc116998804)

[2. Methods 3](#_Toc116998805)

[3. Derivation process for CPL scale-dependence effect of CPL materials: 5](#_Toc116998806)

[4. Discussion of CPL scale-dependence effect of CPL materials 6](#_Toc116998807)

[5. The derivation process for the chiral mirror images material with the same anisotropic size has the opposite sign and equivalent CPL signal 8](#_Toc116998808)

[6. Discussion about measurement entropy 10](#_Toc116998809)

[7. Discussion about potential effect on the polarization state of the incident excitation light 14](#_Toc116998810)

[8. Discussion about the scale-effect model in amorphous organic film PVA 15](#_Toc116998811)

[9. Discussion about the scale-effect model in solution 17](#_Toc116998812)

[10. Schematic diagram of CPL spectrometer 18](#_Toc116998813)

[11. Crystal Data Table 18](#_Toc116998814)

[12. Photographes and reference picture 19](#_Toc116998815)

[13. Angle-dependent CPL spectra of changing the angle between the incident excitation light and the optical axis of the benzil crystal 21](#_Toc116998816)

[14. Angle-dependent experiments of the angle between the constant incident excitation light and the optical axis 25](#_Toc116998817)

[15. Measurement of the entropy 29](#_Toc116998818)

## Experimental Section

**Chemicals**

Cadmium oxide (CdO, 99.99%), zinc acetate dihydrate Zn(Ac_2_)⋅2H_2_O, >99%), selenium powder (99.99%, powder), oleic acid (OA, 90%), octylamine (OAm, 99%), 1-octadecene (ODE, 90%) and tri-n-octylphosphine (TOP, 97%) were purchased from Aldrich. Mercaptopropionic acid (MPA, >99%), tetramethylammonium hydroxide(TMAH) and sulfur powder (99.5%) were purchased from Alfa. All chemicals were used as received without further purification.

**Preparation of Perovskite Polymer Film**

MABr (0.64 mmol), PbBr_2_ (0.8 mmol), and polymer (120mg) was dissolved in DMF under vigorous stirring to form a transparent precursor solution. Then, a mixture solution in DMF (0.1 mL) was deposited onto quartz under ambient air environment via spin-coating at 3000 rpm for 30 s, and annealing at 60 °C of 30 min. during the annealing process. The film gradually turned into green, along with the formation of MAPbBr_3_ NCs.

**Preparation of precursor solution**

Cd stock solutions (0.4 M and 0.1 M) were prepared by dissolving CdO powder in the mixture of OA and ODE (VOA: VODE= 1:1 or 1:4) at 250 °C. The Zn precursor solution (0.1 M) was obtained by dissolving ZnAc_2_ 2H_2_O in the mixture of OAm (1.6 mL) and ODE (18.4 mL) at 140 °C. The S precursor solution (0.1 M) was obtained by dissolving the sulfur powder in dry ODE (20 mL) at 120 °C. The stock solutions above were stored at room temperature.

**Synthesis of water-soluble CdSe/3CdS/2ZnS core/shell/shell QDs**

The water-soluble CdSe/3CdS/2ZnS core/shell/shell QDs (CdSe/CdS/ZnS QDs) were prepared according to the literature procedure. [1-4] The core CdSe QDs were first obtained via a modified hot injection method. Typically, Se powder (0.0017 g, 2 mmol), TOP (0.5 mL) and OAm (4.5 mL) were mixed in a 50 mL three-neck flask. After being degassed for 30 minutes at 90 °C, the mixture was heated to 220 °C in the N_2_ atmosphere to obtain a colorless solution. 0.5 mL Cd stock solution (0.4 M) was injected quickly (in less than 0.2 s) into the above reaction solution. After reacting for 2 min at the temperature at 220 °C, the reaction system was cooled to room temperature. The core CdSe QDs obtained were precipitated and centrifugated by adding ethanol and acetone and redispersed in1 mL of hexanes. The oil-soluble CdSe/CdS/ZnS QDs were then prepared by a classical SILAR (Successive Ion Layer Adsorption and Reaction) method.^5^ Typically, the above core CdSe QDs (1 mL), OA (1.0 mL), and ODE (2.0 mL) were mixed and degassed for 30 min at 90 °C to remove the hexane. The core CdSe QDs solution was further heated to the target temperature in the N_2_ atmosphere to grow the shell layer. Cd and S precursors were alternately injected into above solution at 220 ℃ to form CdS shell, and Zn and S precursor were alternately injected at 200 ℃ to grow ZnS shell. The reaction proceeded until CdSe/CdS/ZnS core/shell/shell QDs with a targeted ZnS shell thickness were obtained. Note that the reaction should be kept for a certain time after each precursor is injected. After the precipitation and centrifugation, the oil-soluble CdSe/CdS/ZnS QDs were modified via the ligand exchange procedure by using bifunctional MPA ligands. [5] Finally, the water-soluble MPA-modified CdSe/CdS/ZnS QDs were obtained and stored for further use.

## Methods

**Photophysical properties of benzil molecule in the rigid matrix:**

The UV-Vis absorption spectra were obtained on a Cary 60 (Agilent Technologies) spectrophotometer. Fluorescence, phosphorescence, and the lifetime of delayed emission spectra were recorded on an Agilent Cary Eclipse spectrophotometer. Phosphorescence mode; Delay time = 0.1 ms; Gate time = 2.0 ms. Photoluminescence spectra were recorded on the HORIBA FluoroMax-4 spectrometer. Absolute PL quantum yields were determined with a spectrometer C11347-11 (Hamamatsu, Japan). Circular dichroism (CD) spectra were acquired using the JASCO J-815 spectrophotometer. CPL spectra were acquired using the JASCO CPL-300 spectrofluoropolarimeter. The measurement mechanism of a CPL spectrometer involves passing spontaneous light waves through a photoelastic modulator to give a fixed phase of the orthogonal components and then through a lock-in amplifier to measure the actual phase difference and amplitude of the spontaneous radiation of materials. Thus, using a CPL spectrometer to validate this idea makes sense, and Fig. S1 shows a brief diagram of the optical test path in the CPL signal measurement in the present study.

Dissolve benzil in a tetrahydrofuran solution, put it into a quartz nuclear magnet tube, and put it into a low-temperature temperature control fitting from Oxford Inc. Add liquid nitrogen to the low-temperature parts of the fitting, freeze it to 77 K, and start the temperature-dependence photophysical spectra experiments.

**Crystal culture of benzil**

The benzil was dissolved in ether solution to prepare a supersaturated solution. The supersaturated solution was filtered through an organic filter membrane (pore size is 0.22 μm) to remove suspended particles in the supersaturated solution. Then, the filtered solution was put into a small bottle, and benzil crystals of different morphologies and sizes were obtained by volatilization. Most of the crystals are regular and orderly, and the crystals can be further polished with sandpaper with 2,000 mesh to 10,000 mesh.

**Preparation of benzil@PVA films**

Dispersed the benzil crystals of different spatial point groups in ethanol with different concentrations (10^-2^ M) and added them to the PVA solution. Afterward, the obtained benzil@PVA solution was coated on a glass sheet, dried by heating (~ 80 °C), and dried in a vacuum to obtain benzil@PVA films of the corresponding configuration.

**Characterization benzil crystals**

First, a micrometer caliper was used to test the distance between different parallel planes of the crystal and use a camera to photograph its topography. Then, the 3D tools in photoshop software were used to build a comparatively accurate three-dimensional (3D) model for elemental analysis.

The benzil crystal was fixed on the high-precision 3D rotating platform. And then the 3D rotating platform was installed on the CPL spectrometer. The normal incidence angle was then determined by the optical path of the transmitted and reflected light. In addition, when the transmitted light transmits through the crystal, the normal incident angle is selected as the incident angle.

**Preparation of PVA films with different CPL signal**

A film puller (Figure S4) to pull up the film, and tested the pulled film in different measurement directions to obtain CPL signals with opposite signs. All the films used in our test are non-recoverable deformable, and the non-recoverable deformable films can be obtained by maintaining the stretching state for 12 hours.

**Integral data processing**

The Numpy package and Pandas package was chosen to process data automatically.

## Derivation process for CPL scale-dependence effect of CPL materials:

It is assumed that the medium is a non-magnetic and transparent medium, which the relative permeability of the medium is $\mu_{0}=1$ and there is no absorption in the band of electromagnetic wave propagation, then it is assumed that the medium is in the macro-small and micro-large volume element of the anisotropic medium. The electric induction intensity in the medium can be described by tensor dielectric function ($\varepsilon_{ik}$).

$$D_{i}=\varepsilon_{ik}E_{k}$$

Where $E_{k}$ is the electric field intensity on the$k$ axis; $D_{i}$ is the electric induction intensity on axis $i$; $\varepsilon_{ik}$ is the tensor dielectric function in the medium.

If the following equations are combined:

$$\left\{ \begin{aligned} \vec{D_{i}}=\varepsilon_{ik}\vec{E_{k}} \\ \vec{H}=\vec{n}\times\vec{E} \\ \vec{D}=-\vec{n}\times\vec{H} \end{aligned} \right.$$

For the three components of vector $\vec{E}$, homogeneous equations can be constructed:

$$\begin{aligned} \left( n^{2}\cdot\delta_{ik}-n_{i}n_{k}-\varepsilon_{ik} \right)E_{k}=0\#(S1) \end{aligned}$$

The expansion of formula 1 is obtained[6],

$$n^{2}\left( \varepsilon^{\left( x \right)}n_{x}^{2}+\varepsilon^{\left( y \right)}n_{y}^{2}+\varepsilon^{\left( z \right)}n_{z}^{2} \right)-[n_{x}^{2}\varepsilon^{\left( x \right)}\left( \varepsilon^{\left( y \right)}+\varepsilon^{\left( z \right)} \right)+n_{y}^{2}\varepsilon^{\left( y \right)}\left( \varepsilon^{\left( x \right)}+\varepsilon^{\left( z \right)} \right)+n_{z}^{2}\varepsilon^{\left( z \right)}\left( \varepsilon^{\left( x \right)}+\varepsilon^{\left( y \right)} \right)]+\varepsilon^{\left( x \right)}\varepsilon^{\left( y \right)}\varepsilon^{\left( z \right)}=0$$

Where, for monochromatic electromagnetic waves, $\varepsilon_{\left( \omega\right)}^{x}$*,* $\varepsilon_{\left( \omega\right)}^{y}$ and $\varepsilon_{\left( \omega\right)}^{z}$are constants. Then, when $\hat{n}$ is certain, the equation above is a quadratic equation of $n^{2}$ with two real solutions $n_{1}$ and $n_{2}$. It is proved that there exist two refractive indices $n_{1}\neq n_{2}$ of the intrinsic electromagnetic waves with independent polarization states in the anisotropic medium with the direction $\hat{n}$, and the corresponding electric field induction intensities $\vec{D_{1}}$ and $\vec{D_{2}}$ respectively, i.e

$$\vec{D_{1}}\cdot\vec{D_{2}}=0$$

For the electromagnetic wave under this condition, the wave vector $k =\frac{\omega}{c}n$, and $k_{1}\neq k_{2}$, namely:

$$\left\{ \begin{aligned} \vec{k_{1}}=k_{1}\cdot\hat{k} \\ \vec{k_{2}}=k_{2}\cdot\hat{k} \end{aligned} \right.$$

The phase difference$\Delta\varphi$ between $\vec{D_{1}}$ and $\vec{D_{2}}$ of the transmission distance $L$ in the medium can be described as follows:

$$\frac{\lambda}{2\pi}\left| \Delta\varphi\right|=\frac{\lambda}{2\pi}\left| \varphi_{1}-\varphi_{2} \right|=\frac{\lambda}{2\pi}\cdot c\cdot\left| n_{1}-n_{2} \right|\cdot\frac{L}{c}=\left| n_{1}-n_{2} \right|\cdot L$$

When phase difference $\left| \Delta\varphi\right|$ is far less than the cycle of electromagnetic wave, the wave of phase can be ignored, there are:

$$\left| n_{1}-n_{2} \right|\cdot L\ll\lambda$$

Then transform the inequality, and get:

$$L\ll R=\lambda\left| \frac{1}{n_{2}-n_{1}} \right|$$

## Discussion of CPL scale-dependence effect of CPL materials

The effect of the anisotropic medium of the environment on the generation of elliptically polarized waves can be observed to highly depend on the wavelength of the electromagnetic waves passing through the medium and the magnitudes of the refractive indices ($n_{1}$,$n_{2})$ of the anisotropic medium in the propagation direction. When $L$ is finite and $n_{1}\sim n_{2}$ in the anisotropic medium, the phase difference between $\vec{D_{1}}$ and $\vec{D_{2}}$ induced by the anisotropic medium is negligible. Considering that the emission band for circularly polarized luminescent materials is mainly in the visible region and near-infrared region, the luminescent band from 390 nm ~ 900 nm is first considered. This model is first discussed in the context of liquid crystal materials to evaluate whether this model is adaptable to the experimentally observed polarization optics [7]. According to reported data [8], such liquid crystal materials have $n_{1}=\sqrt{\varepsilon_{\parallel}}$ and $n_{2}=\sqrt{\varepsilon_{\perp}}$ at room temperature, with reference to Fig. S2 for the data.

Therefore, the impact of the liquid crystal medium on the phase difference of orthogonally polarized electromagnetic waves $\vec{D_{1}}$ and $\vec{D_{2}}$ transmitted in the $\hat{n}$ direction can be easily analyzed based on the above model. At a temperature of 20°C, the anisotropy scale ($L$) of the homogeneous anisotropic medium is much smaller than a 1-micron order of magnitude. At 35°C, the anisotropic effect of the liquid crystal anisotropic medium on the electromagnetic wave phase can be ignored only when the anisotropy scale of the uniform anisotropic medium is much smaller than a 100-micron order of magnitude. This is in accordance with the current conclusion regarding the CPL signal induced by liquid crystal materials: a liquid crystal is orderly arranged at room temperature, which effectively induces CPL. With increasing temperature, the arrangement of liquid crystal molecules becomes chaotic, and the isotropic properties become more obvious. Therefore, a longer anisotropy scale in the uniform medium is required to affect the phase difference between the two electromagnetic waves in the same $\hat{n}$ direction. With a further increase in the isotropy, $n_{1}\approx n_{2}$, the phase difference induced by the anisotropic medium under the same optical path becomes increasingly difficult to observe, showing a macroscopic isotropy property.

Taking naturally occurring amino acids as examples, for the reliable data of naturally occurring amino acids can be consulted in the manual [9]. Organic molecules basically have larger structures than naturally occurring amino acids if a single organic molecule can emit light. The van der Waals volume of naturally occurring amino acids is approximately 0.1~0.2 $nm^{3}$[9]. If these small molecules are assumed to be cubes, then the cube diameter is approximately 0.5 to 0.6 nm. The molecular size of small amino acid molecules must be greater than 0.5~0.6 nm, and the diameter of larger organic luminescent molecules that can spontaneously emit radiation could also be greater than 0.6 nm. Under this dielectric condition, if the organic molecules orderly assemble or aggregate, then the phase difference caused by the homogeneous anisotropic medium is not negligible. Only under strict monomolecular conditions can the luminescence properties in isotropic media be considered to analyze the effect of the intrinsic chirality of excited states on the CPL emission. This order of magnitude analysis is also consistent with the dielectric distribution of anisotropic media reported in the literature [10].

## The derivation process for the chiral mirror images material with the same anisotropic size has the opposite sign and equivalent CPL signal

At present, molecular stacking materials with spiral assembly structures or supramolecular assembly materials are widely used in academics to develop luminescent materials with CPL emissions. In this paper, the scale-dependence CPL model based on anisotropic media is used to prove that in anisotropic media with chiral mirror images structure (dielectric distribution of enantiomer), the chiral mirror images material with the same anisotropic size has the opposite sign and equivalent CPL signal.

It is assumed that there are pairs of chiral mirror images of anisotropic materials: LM (Left-handed mateiral) and RM (Right-handed mateiral), whose corresponding dielectric tensors are$\varepsilon_{ik}$ and ${\varepsilon'}_{ik}$ respectively. Its corresponding dielectric spindles exist in space are $\varepsilon^{\left( x \right)}, \varepsilon^{\left( y \right)},\varepsilon^{\left( z \right)}$and ${\varepsilon'}^{\left( x \right)}, {\varepsilon'}^{\left( y \right)},{\varepsilon'}^{\left( z \right)}$ respectively. Then the dielectric tensors of LM and RM can be expressed as:

$$\varepsilon_{ik}=\left( \begin{matrix} \varepsilon^{\left( x \right)} & 0 & 0 \\ 0 & \varepsilon^{\left( y \right)} & 0 \\ 0 & 0 & \varepsilon^{\left( z \right)} \end{matrix} \right)$$

$${\varepsilon'}_{ik}=\left( \begin{matrix} {\varepsilon'}^{\left( x \right)} & 0 & 0 \\ 0 & {\varepsilon'}^{\left( y \right)} & 0 \\ 0 & 0 & {\varepsilon'}^{\left( z \right)} \end{matrix} \right)$$

Because the assembly or stacking of material molecules is enantiotopic, the other properties of the ordered structure are the same. Therefore, it can be determined that the moduli of the dielectric function of LM and RM are the same, wherein:

$$\left| \varepsilon_{ik} \right|=|{\varepsilon'}_{ik}|$$

Since LM and RM materials are assembled or stacked in enantiotopic, assuming that their assembly state is spiral, it is easy to find that the long axis of spiral assembly is unchanged along this dielectric spindles axis, wherein:

$$\varepsilon^{\left( z \right)}{=\varepsilon'}^{\left( z \right)}$$

On this basis, if the dielectric distribution of both is enantiotopic, the following conditions are required:

$$\varepsilon^{\left( x \right)}= {\varepsilon'}^{\left( y \right)}$$

$$\varepsilon^{\left( y \right)}= {\varepsilon'}^{\left( x \right)}$$

Then the dielectric distribution of LM and RM materials meets the enantiomorphism condition (chiral enantiomorphism). Then, in the anisotropic medium condition, in the same propagation direction $\hat{n}$, and plug in the following equation,

$$n^{2}\left( \varepsilon^{\left( x \right)}n_{x}^{2}+\varepsilon^{\left( y \right)}n_{y}^{2}+\varepsilon^{\left( z \right)}n_{z}^{2} \right)-[n_{x}^{2}\varepsilon^{\left( x \right)}\left( \varepsilon^{\left( y \right)}+\varepsilon^{\left( z \right)} \right)+n_{y}^{2}\varepsilon^{\left( y \right)}\left( \varepsilon^{\left( x \right)}+\varepsilon^{\left( z \right)} \right)+n_{z}^{2}\varepsilon^{\left( z \right)}\left( \varepsilon^{\left( x \right)}+\varepsilon^{\left( y \right)} \right)]+\varepsilon^{\left( x \right)}\varepsilon^{\left( y \right)}\varepsilon^{\left( z \right)}=0$$

For LM and RM materials, there are two different real solutions of the corresponding refractive index, $n_{1}$ and $n_{2}$, and there is a corresponding relationship between the two real solutions.

Based on this, suppose the value of the refractive index of LM material are $n_{1,LM}=a,n_{2,LM}=b$ relatively; thus $n_{1,RM}=b,n_{2,RM}=a$ are the refractive index value of RM materials.

For the CPL test in the experiment, it can be assumed that the sample scale are the same, that is, $L=L_{LM}=L_{RM}$.

The phase difference formula in the anisotropic scale model can be obtained as follows:

$$\Delta\varphi_{LM}=\frac{\left( n_{2,LM}-n_{1,LM} \right)}{n_{1,LM}n_{2,LM}}\cdot L_{RM}=(b-a)\cdot L$$

$$\Delta\varphi_{RM}=\frac{\left( n_{2,RM}-n_{1,RM} \right)}{n_{1,RM}n_{2,RM}}\cdot L_{RM}=(a-b)\cdot L$$

It's easy to conclude as follows:

$$\Delta\varphi_{LM}+\Delta\varphi_{RM}=0$$

If the assembled structures of samples with the same scale are corresponding and their dielectric distribution is also assumed to be corresponding, then the phase difference between the two waves in LM and RM media in the same direction are negatives to each other in the enantiomer. The phase difference is the absolute factor affecting the CPL spectral signal, and the test results in the CPL spectrometer are (+)-CPL emission and (-)-CPL emission. By the way, the positive and negative CPL signals are determined by the testing conditions of the instrument and the testing software.

## Discussion about measurement entropy

Considered a range of electromagnetic wave $\vec{D}$ propagation in an anisotropic medium. In its initial state, $\vec{D}$ would decomposition into two columns component with the orthogonal electric vector $\vec{D_{1}}$ and $\vec{D_{2}}$ .

$$\vec{D}=\vec{D_{1}}+\vec{D_{2}}$$

The wave function of $\vec{D_{1}}$ and $\vec{D_{2}}$ are as follows.

$$\vec{D_{1}}=\vec{D_{1,0}}\exp i\left( \vec{k}\cdot\vec{r}-\omega t+\phi_{1} \right)$$

$$\vec{D_{2}}=\vec{D_{2,0}}\exp i\left( \vec{k}\cdot\vec{r}-\omega t+\phi_{2} \right)$$

$\vec{D_{1}}$ and $\vec{D_{2}}$ have the same initial phase. When $\vec{D_{1}}$ and $\vec{D_{2}}$ propagation in an anisotropic medium, the phase difference $\Delta\varphi$ would generate due to the difference in velocity of propagation of $\vec{D_{1}}$ and $\vec{D_{2}}$.

$$\Delta\varphi=\varphi_{1}-\varphi_{2}$$

In the condition of vacuum or atmosphere, when the phase difference of $\vec{D_{1}}$ and $\vec{D_{2}}$ is the odd number of times of $\pi/2$, $\vec{D}$ is CPL, and when the phase difference of $\vec{D_{1}}$ and $\vec{D_{2}}$ is the even number of times of $\pi/2$, $\vec{D}$ is the LPL. And the light with other phase differences is elliptically polarized light (EPL). Wherein, CPL and LPL could be considered EPL with specific phase differences.

Take advantage of the formula to obtain above to calculate the phase difference of light generated by the scale effect, the specific criteria of medium scale to generate CPL and LPL could be quantization. The formula to calculate the phase difference from the scale effect is as follows.

$$\Delta\varphi=\frac{2\pi\cdot L\cdot\left( n_{1}-n_{2} \right)}{\lambda}$$

For circular polarization light, the phase difference of $\vec{D_{1}}$ and $\vec{D_{2}}$ should be the odd number of times of $\pi/2$ as the following criteria.

$$\frac{2\pi\cdot L_{CPL}\left( n_{1}-n_{2} \right)}{\lambda}=\frac{\left( 2n-1 \right)}{2}\pi$$

For LPL, the phase difference of $\vec{D_{1}}$ and $\vec{D_{2}}$ should be an even number of times of $\pi/2$ as the following criteria.

$$\frac{2\pi\cdot L_{LPL}\left( n_{1}-n_{2} \right)}{\lambda}=n\pi$$

Then, the following criteria could be obtained.

$$\begin{aligned} \left\{ \begin{matrix} L_{LPL}=\frac{\left( 2n-1 \right)}{4}\lambda\cdot\frac{1}{n_{1}-n_{2}} \\ L_{CPL}=\frac{n}{2}\cdot\frac{1}{n_{1}-n_{2}} \\ L_{\begin{matrix} LPL\to CPL \\ CPL\to LPL \end{matrix}}=\frac{1}{4}\cdot\frac{1}{n_{1}-n_{2}} \end{matrix} \right.\#(S2) \end{aligned}$$

Wherein, $L_{LPL}$ is the distance of spontaneous radiation conversion into LPL, $L_{CPL}$ is the distance that conversion into CPL, and $L_{\begin{matrix} LPL\to CPL \\ CPL\to LPL \end{matrix}}$ is the required distance that CPL (or LPL) could transform into LPL (or CPL).

First, the simplest case was considered here. When the resulting phase difference $\Delta\varphi\in\left( 0,\pi\right]$, which is $n=1$,

$$\begin{aligned} L_{CPL}=\frac{1}{4}\lambda\frac{1}{n_{1}-n_{2}}\#\left( S3 \right) \end{aligned}$$

$$\begin{aligned} L_{LPL}=\frac{1}{2}\lambda\frac{1}{n_{1}-n_{2}}\#\left( S4 \right) \end{aligned}$$

Next, the properties of polarized light can be initially quantified by considering the proportion of CPL and LPL compared to the proportion of polarized light in a general EPL. Because of the periodic property of electromagnetic waves, it is sufficient to consider the ratio of CPL and LPL to EPL in one period.

It is easy to analyze with simple calculus knowledge. For a particular wavelength, the $\Delta\varphi$ change could be defined as the measurement accuracy of the spectrometer with minimal accuracy to resolve the change of the phase difference $d\varphi$. The following relationships exist:

$$\begin{aligned} Nd\varphi=2\pi\#\left（ S5 \right） \end{aligned}$$

Then a period of phase difference is divided into $d\varphi$ parts by the resolution of the spectrometer $N$, each of which corresponds to a value that can be measured by the spectrometer to detect the variable phase difference.

It is easy to define the $\xi$ as the ratio function of CPL and LPL relative to EPL:

$$\begin{aligned} \xi=\frac{4\cdot d\psi}{\left( N-4 \right)d\psi}=\frac{4}{N-4}\#\left（ S6 \right） \end{aligned}$$

Wherein, the value 4 in equation (S6) is means the special divisions where the $\Delta\varphi$ is the interger multiples of $\frac{\pi}{2}$ ($\Delta\varphi=\frac{\pi}{2},\pi,\frac{3\pi}{2},2\pi$). Therefore, it is only necessary to determine the size of the resolution of the spectrometer for the phase difference to judge the ratio of CPL and LPL to EPL.

For the CPL spectrometer, the transverse coordinates of the CPL spectrum are wavelengths (nm), and the units of the longitudinal coordinates are millidegree (mdegs). According to the instruction of the CPL spectrometer, they measure the signal of polarized light by the phase difference measurement method by using the lock-in amplifier.

It is easy to discover that the background noise of the CPL spectrometer is affected by the shape distribution of the sample. The CPL spectrometer must have different background noise for the light ejected from the powder and the light ejected from the crystal. The order of background noise of the phase difference measured by the CPL spectrum can be determined by the comparison of the order of baseline.

The CPL signal of naphthol derivative in solution state with recognized CPL signal was selected for analysis, and it was found that the order of magnitude of baseline for the solution-phase CPL spectrum was around 0.01 mdeg. In order to eliminate the influence of the baseline fluctuation on the instrument, it is necessary to take two orders of magnitude more accuracy to ensure that the maximum error of the instrument is less than 10%. Assuming that 1 mdeg is a minimum background noise of the CPL spectrometer, the following conclusion could be obtained by converting the angle expression into radius expression.

$$N\cong\frac{2\pi}{dx}=\frac{2\pi\times360\times1000}{0.1\times2\pi}=3.6\times{10}^{6}$$

$$\begin{aligned} \xi\cong\frac{4}{3.6\times{10}^{6}-4}=1.1\times{10}^{-6}\to0\#\left（ S7 \right） \end{aligned}$$

Therefore, it is easy to discover that the polarization signals we measured were mainly general EPL, whereas the contribution of CPL and LPL was essentially none.

For the ratio function $\xi$, the conditions that could measure the CPL and LPL are also included. When the resolution of the instrument is extremely low (when N is small), there is a high probability to measure the CPL and LPL signals. For instance, the signals of CPL and LPL were measured to account for $\xi=66\%$ of the EPL when N = 10. (Note: If calculated according to the absolute accuracy of the CPL spectrometer ~${10}^{-6}$ mdeg of the CPL spectrometer, the same conclusion was obtained $\xi\to0$)

If the main measurement object of the CPL spectrometer is EPL, it is easy to realize the view that the entropy$S$ of the measurement system is the main factor affecting the homogeneity and heterogeneity properties of the polarized signal. The statistical definitions of entropy are defined as follows:

$$S=k_{B}\ln\Omega$$

where $k_{B}$ is the Boltzmann constant, $\Omega$ is the equal probability state.

In a single CPL measurement, they simultaneously have only one state measured $\Omega_{Single}$ for crystals or films; however, the measured state is related to the number of aggregates $\Omega_{Multi}$ in the light spot for the dispersed states such as solution or powder. There is $\Omega_{Multi}>>\Omega_{S\mathrm{ingle}}$.

Then, for the system of $\Omega_{Single}$, if the measured object is EPL. Suppose an ellipse with the long axis length $2a$ and the short axis length $2b$ in the condition of $a>b$ (as shown in the following Fig.S12 on the left). Then the signal of EPL is anisotropic for the CPL spectrometer. Assuming that the CPL spectrometer using short-axis 2b of an elliptical as the measurement origin, the phase difference in the direction of the long axis 2a concerning the long axis 2b is $\Delta\varphi=\varphi_{2a}-\varphi_{2b}$. If the CPL spectral measurement direction is changed, the elliptical long axis 2a becomes the measurement origin of the instrument, and the phase difference of the instrument test becomes the short axis 2b relative to the long axis 2a $\Delta\varphi'=\varphi_{2b}-\varphi_{2a}$. It is obvious $\Delta\varphi+\Delta\varphi'=0$. The same sample measured separately for axis 2a and 2b as the measured origin could theoretically obtain the opposite signal, which is the anisotropic signal.

And for the system of $\Omega_{Multi}>>\Omega_{Single}$, each state produces polarized light just like the system of $\Omega_{Single}$. Since the instrument detects the light of one certain detection plane at a time, the $2a$ distribution of the elliptical long axis can be considered on the plane. Since the major axis 2a of the ellipse is orthogonal to the minor axis 2b of the ellipse, the situation of the corresponding minor axis can be known by considering the major axis situation. According to the equipartition theorem, the direction of the elliptical long axis vector in the plane of the isotropy should be evenly distributed in the plane, then in the space, there have angle elements $\vartheta=\frac{2\pi}{\Omega}$. Then, under the condition of $\Omega_{Multi}>>\Omega_{Single}$, there are:

$$\lim_{\Omega\to\infty} \vartheta=0$$

This as shown on the right side of the Fig.S12 below. The short axis also satisfies this constraint.

Then for the CPL spectrometer, the signal was measured under the conditions $\Omega_{Multi}>>\Omega_{Single}$ is isotropic.

## Discussion about potential effect on the polarization state of the incident excitation light

At present, it is shown that a medium with Fluorescence Mueller matrix $\boldsymbol{F}\left( \mathbf{d} \right)$ whose differential Mueller matrix $\boldsymbol{M}_{\boldsymbol{e}}$ of emission wavelength continuously changes in the light propagation direction $z$ can be described by the following formula [11]:

$$\boldsymbol{F}\left( \mathbf{d} \right)=\mathbf{M}_{e}(L)\int_{0}^{L} \boldsymbol{M}_{\boldsymbol{e}}^{-\mathbf{1}}\left( z \right)\boldsymbol{S}\left( z \right)\boldsymbol{M}_{\boldsymbol{x}}\left( z \right)dz$$

$$= \int_{0}^{d} \left. exp(\left( L-z \right)\mathbf{m}_{\text{e}} \right) \mathbf{S}\exp(z\mathbf{m}_{\text{x}})\text{d}z$$

Where $\boldsymbol{M}_{\boldsymbol{e}}$ and $\boldsymbol{M}_{\boldsymbol{x}}$ are both Muller matrices, which are used to describe the propagation quantity of emission light and excitation light in anisotropic structures, respectively; $\boldsymbol{M}_{\boldsymbol{e}}$ and $\boldsymbol{M}_{\boldsymbol{x}}$ are differential Muller matrices at the emission wavelength and the incident wavelength, respectively, and are differential forms of $\boldsymbol{M}_{\boldsymbol{e}}$ and $\boldsymbol{M}_{\boldsymbol{x}}$; $L$ is the length of the medium, and $z$ is the length of the incident light propagating in the medium; $L-z$ is the propagation length of the emitted light in the medium. It can be found that the propagation of incident light in the medium is also affected by the scale effect, and the polarization state of incident light may affect the polarization state of emitted light [12, 13]. However, due to the lack of relevant experimental studies, it is difficult to analyze the influence of the scale effect of incident light on the polarization signal of spontaneous emission.

## Discussion about the scale-effect model in amorphous organic film PVA

Phenomenally, RhB exhibits aggregation-induced quenching and can emit strong photoluminescence at a lower concentration. Therefore, the anisotropy scale of RhB is small under the condition of good dispersion. At present, intrinsic CPL emission by RhB molecules has not been shown, but CPL emission of RhB induced by chiral media or supramolecular assembly has been reported [14-16]. Therefore, RhB satisfies the constraint of $L_{aggregate}\ll R$. The photoluminescence wavelength of CdSe QDs varies with the particle size. The average particle size of CdSe QDs with an emission wavelength of 610 nm is approximately 2.4 nm. Control experiments show that the light emitted by the CdSe QD film is natural light, and a CPL signal cannot be detected by the instrument. Thus, the CdSe QD film can be judged to satisfy the constraint of $L_{aggregate}\ll R$. Fig. 2a~2d shows opposite CPL spectra of RhB@PVA under different tensile conditions. As shown in Fig. 2e~2h, under different tensile conditions, the CPL spectra of CdSe QDs@PVA show CPL signals with opposite signs. $L_{anisotropic medium}\geq R$ could be provided by anisotropic changes in the external environment of the luminescent material, and $L=L_{aggregate} +L_{anisotropic medium}\geq R$. This process can effectively change the CPL signal of the spontaneous emission of luminescent materials, which is unrelated to the structural characteristics of the luminescent molecules. In addition, tetraphenylethene (TPE) molecules [17, 18] with a different luminescence mechanism from RhB and QDs were selected for study. TPE molecules spontaneously assembled in the bad solvent, and luminescence in the aggregated state was observed (Fig. 2i~2l). Under the condition of good dispersion, the fluorescence of TPE will be quenched due to photochemical reactions [19]. Because the TPE molecule has good solubility in organic solvents, it will spontaneously aggregate in the film. The control experiment showed that there was no CPL emission of TPE in the PVA film. When the same tensile anisotropy is introduced into the film, the luminescence intensity and CPL emission increase with increasing TPE concentration. The CPL signal measured in the experiment is further demonstrated to feed back the effect of the material on the environment where the luminescent molecule is located, rather than the effect of the material molecule itself. Perovskite-based luminescent films (λ_ex_ = 365 nm, λ_em_ = 520 nm) were also prepared. The perovskite materials rapidly degrade in high-polarity solvent environments, so doping them into PVA films to generate an anisotropic environment is difficult. An anisotropic transparent PVA film was added into the optical path between the detector and the luminescent sample. Fig. 2m-2p shows that the initial perovskite-based luminescent film does not have a CPL signal without an anisotropic environment, whereas anisotropic environment-dependent CPL signals were detected when the anisotropic PVA film was added into the optical path. Fig. 2m-2p shows the opposite CPL signals of the perovskite-based composite films.

## Discussion about the scale-effect model in solution

For small organic molecules dispersed in a solution, the size of the aggregates at lower concentrations is determined only by the thermodynamic minimum. In this case, the concentration of small organic molecules is proportional to the concentration of aggregates in the solution. Based on formula (1), when the particle size of anisotropic aggregates generated by assembly or stacking of luminescent molecules is $L\geq R$, the change in the CPL signal caused by the anisotropy of the medium cannot be ignored. When $L\geq R$, the spontaneous radiation generated by the dispersed aggregates in the solution will generate a CPL signal. However, when the molecular concentration is low, the concentration of the aggregates in the solution is very low, and the CPL signal from the aggregates is lower than the detection limit of the CPL spectrometer; thus, no CPL signal is detected. When the concentration of molecules is high, the concentration of aggregates in the solution is high, and a CPL signal is detected. We chose a binaphthol derivative ((S/R)-2,2’-diethoxy-1,1’-binaphthyl) that is reported in the literature [20, 21] as not having a Tyndall effect in solution and as having a clear solution state CPL signal for a concentration-dependent CPL spectroscopy study. The experiment showed that the CPL signal of the system gradually decreased with decreasing concentration of the luminescent molecules, and the CPL signal even disappeared when the concentration dilution reached 10^-5^ mol/L (Fig. 2q~2t). Additionally, related studies have used the difference between single-molecule luminescence and excimer luminescence of organic molecules to find that the spontaneous radiation from the single-molecule solution state does not have a CPL signal, whereas the spontaneous radiation from the multiple-molecule aggregated solution state has a CPL signal [20, 21]. The scale-effect model could be another choice here to explain these phenomena simple and clear.

## Schematic diagram of CPL spectrometer


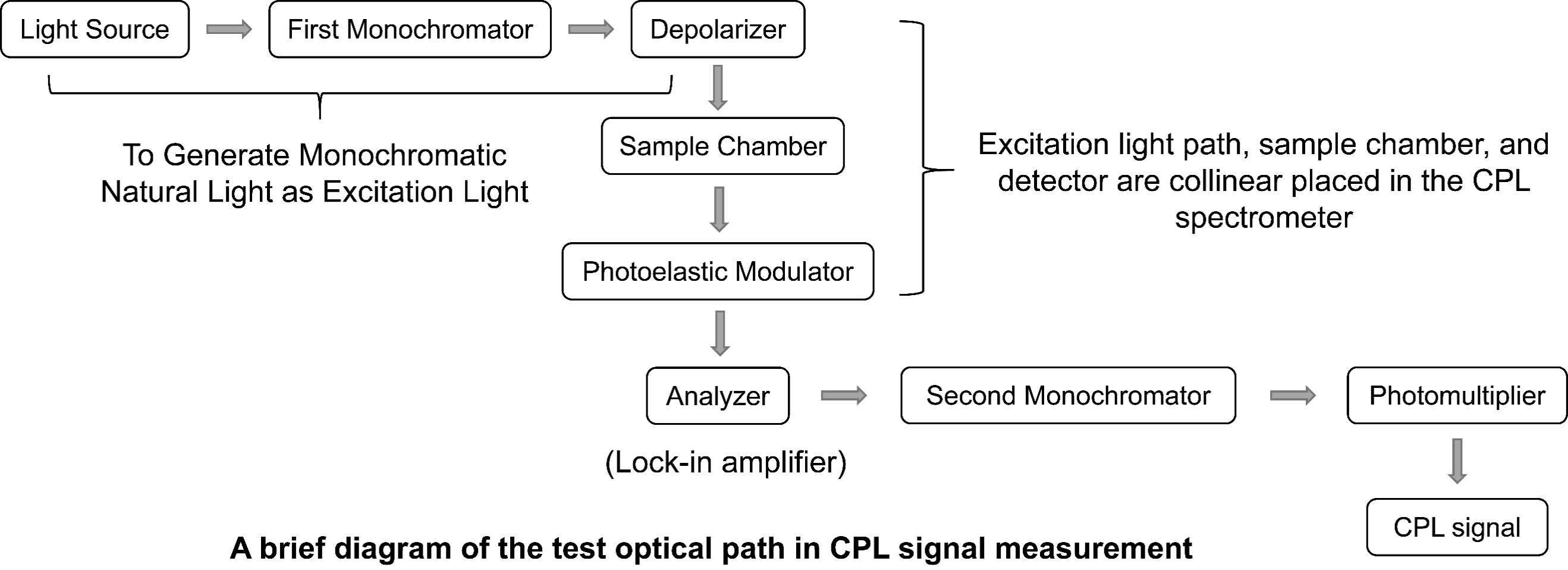


**Figure S1.** A brief diagram of the optical test path in CPL signal measurement. ^6^

## Crystal Data Table

|  | **Benzil-1** | **Benzil-2** |
| --- | --- | --- |
| Space Group | P3121 | P3221, |
| Cell | a 8.409Å  b 8.409Å  c 13.677  α 90°  β 90°  γ 120° | a 8.41Å  b 8.41Å  c 13.672Å  α 90°  β 90°  γ 120° |
| Formula | C14 H10 O2 | C14 H10 O2 |
| Temperature | 297 K | 295 K |
| Deposition Number | 2208319 | 2208320 |

**Table S1** Single crystal data of Benzil.

## Photographes, reference picture, and photophysics data

**
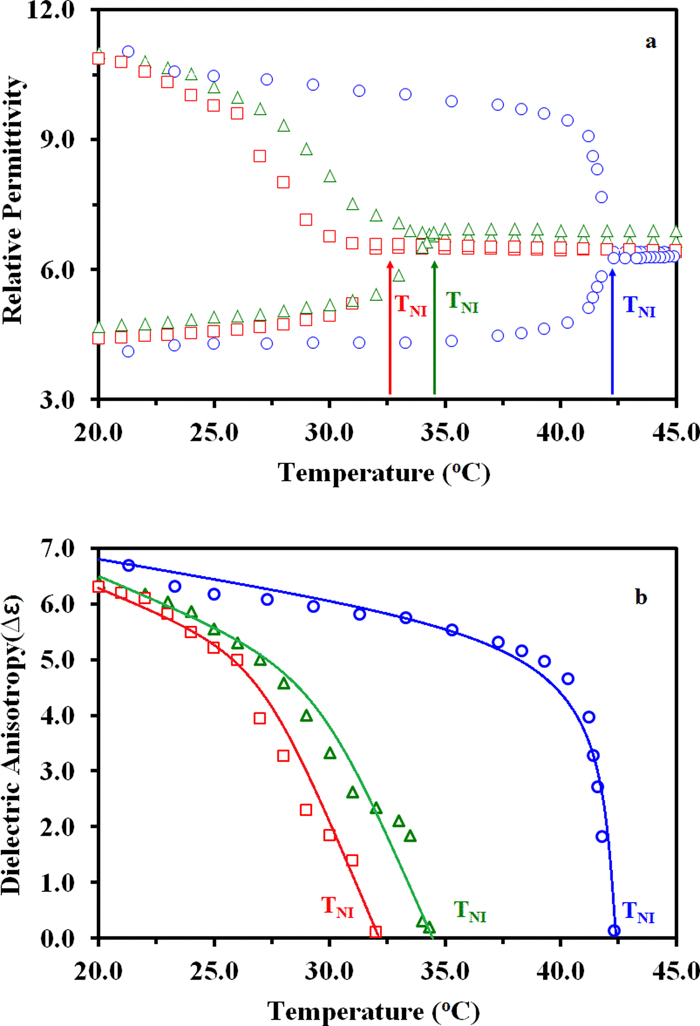
**

**Figure S2.** Dielectric properties of liquid crystal materials reported in the literature. ^7^

**
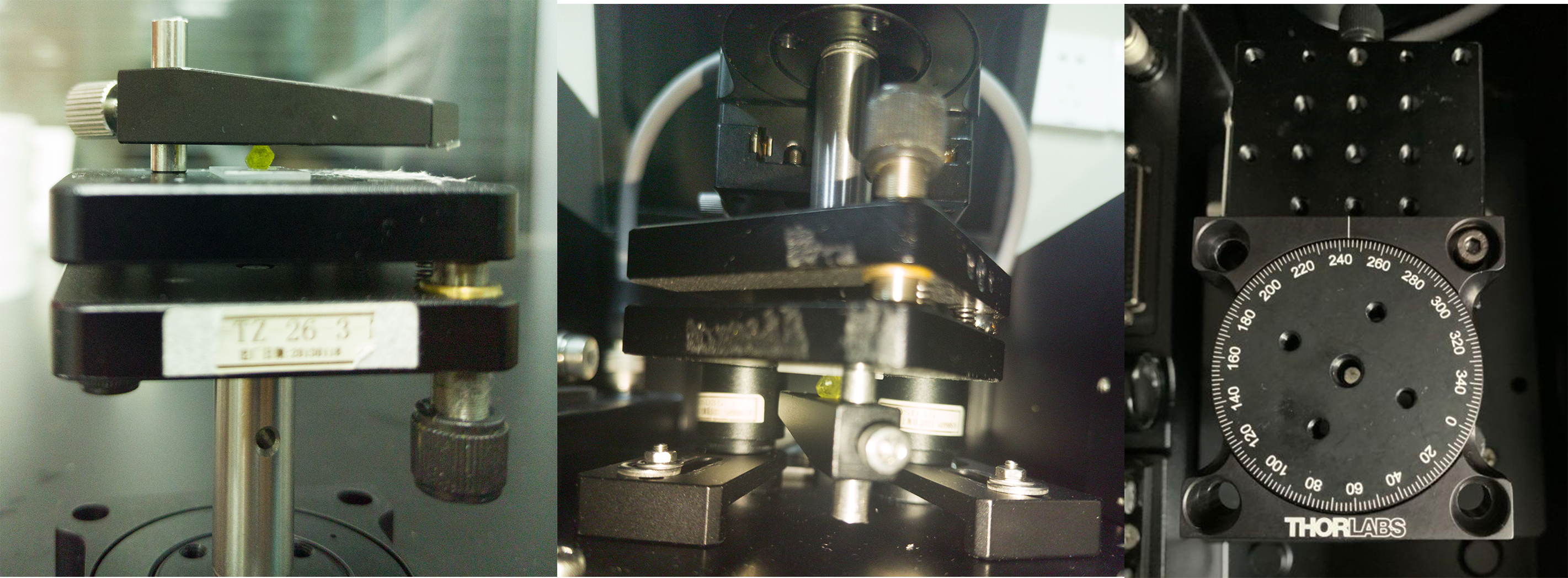
**

**Figure S3.** Photo of the rotating platform


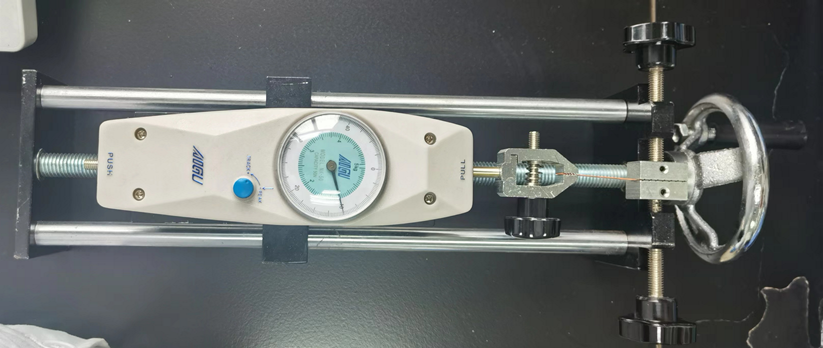


**Figure S4.** Photo of the film puller.


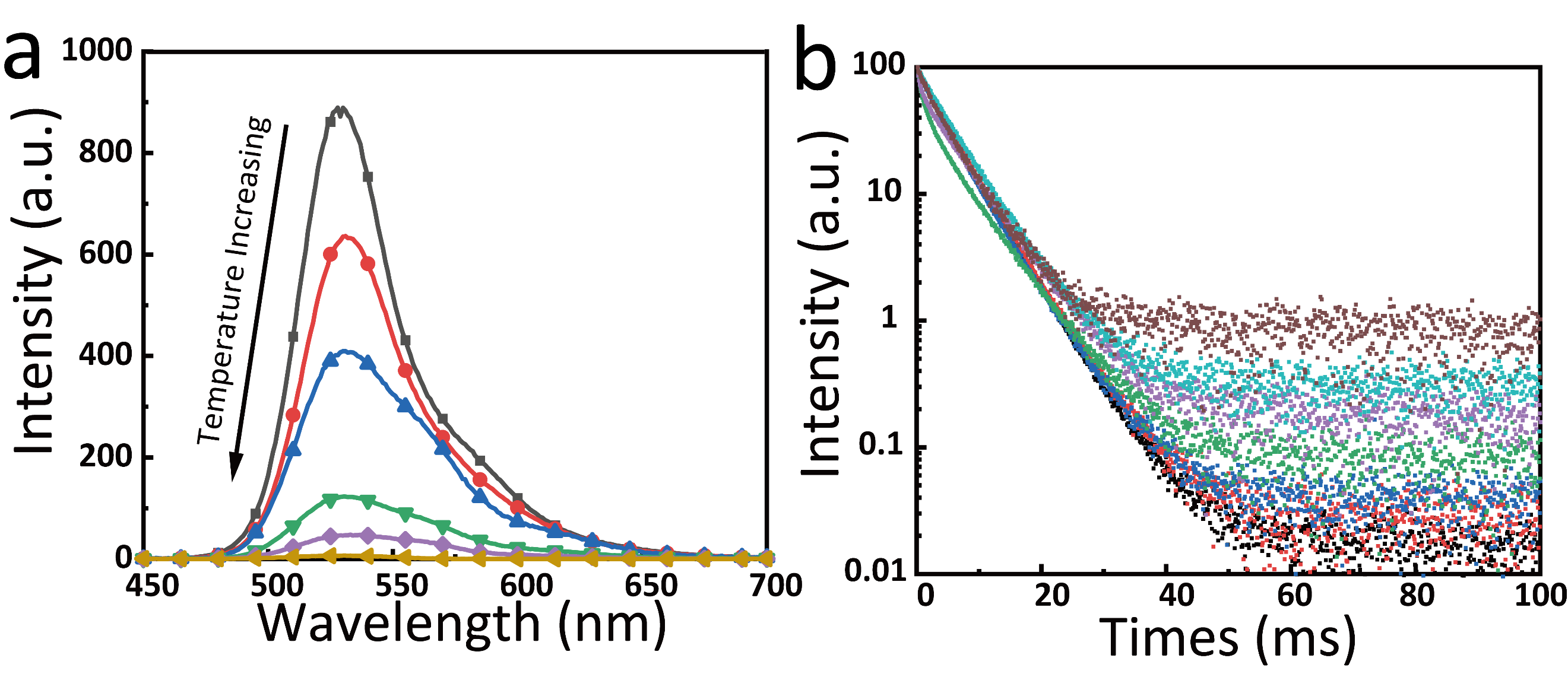


**Figure S5.** Figure S2.The delayed spectra (a) and the lifetime spectra (b) of benzil in glassy solvent (2-methyltetrahydrofuran)from 77 K to 137 K

## Angle-dependent CPL spectra of changing the angle between the incident excitation light and the optical axis of the benzil crystal

The following figures were the angle-dependent CPL spectra of changing the angle between the incident excitation light and the optical axis of the benzil crystal. Here, the wavelength of the incident excitation light was 360 nm, the incident direction of 142° was the incident angle parallel to the optical axis direction of the crystal, and the wavelength detected by the detector was 530 nm.


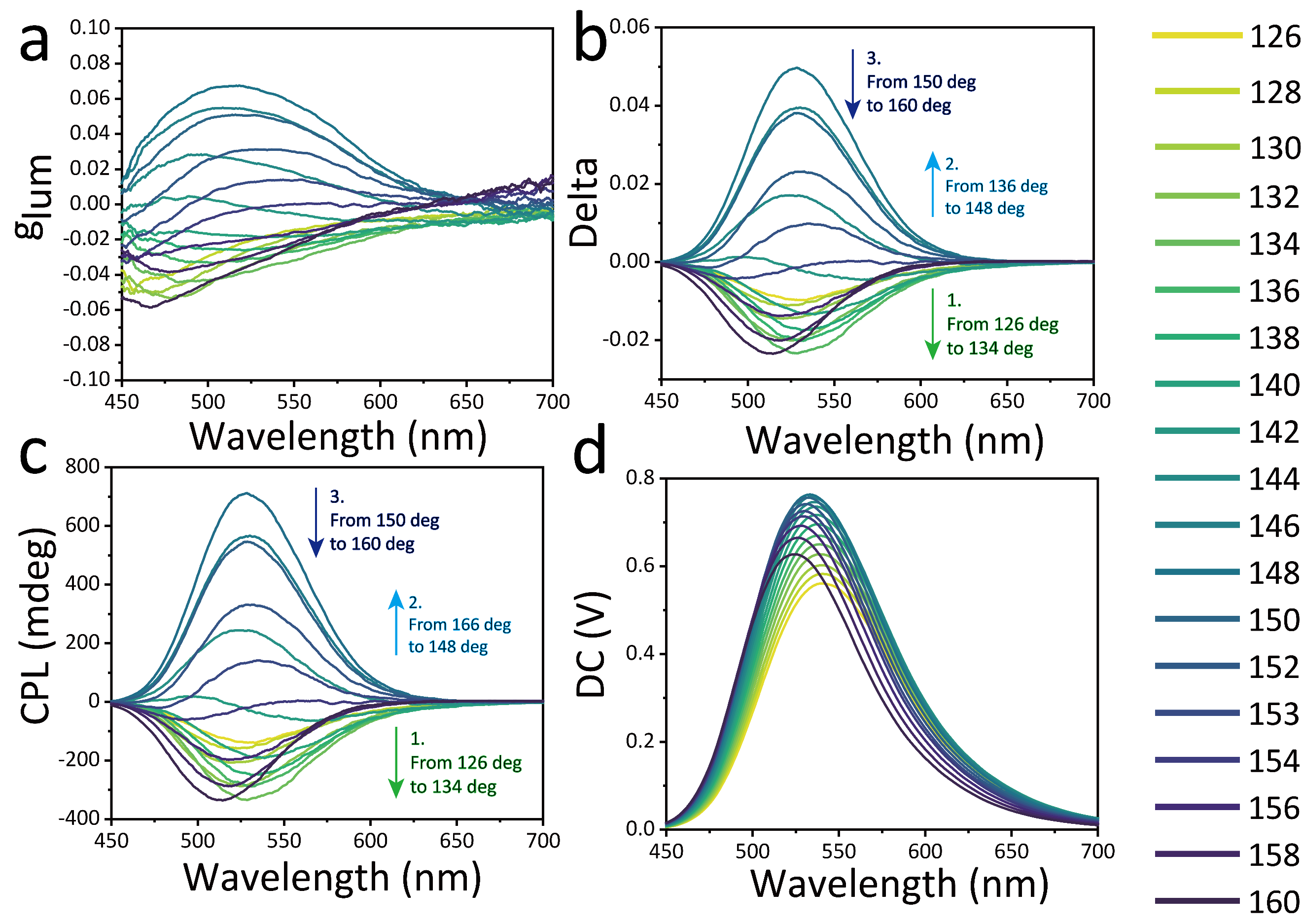


**Figure S6.** The direction of 142° in the figure was the angle of normal incidence of the excitation light onto the model benzil crystal face. In this case, the direction at 142° was parallel to the optical axis direction of the crystal. This figure shows the spectra of the change in CPL signal after changing the incidence angle of the excitation light (from 126° to 160°) when the wavelength of the incident excitation light was 360 nm. (a) the g_lum_ value spectra, (b) the delta value spectra, (c) the CPL spectra, and (d) the light intensity spectra.


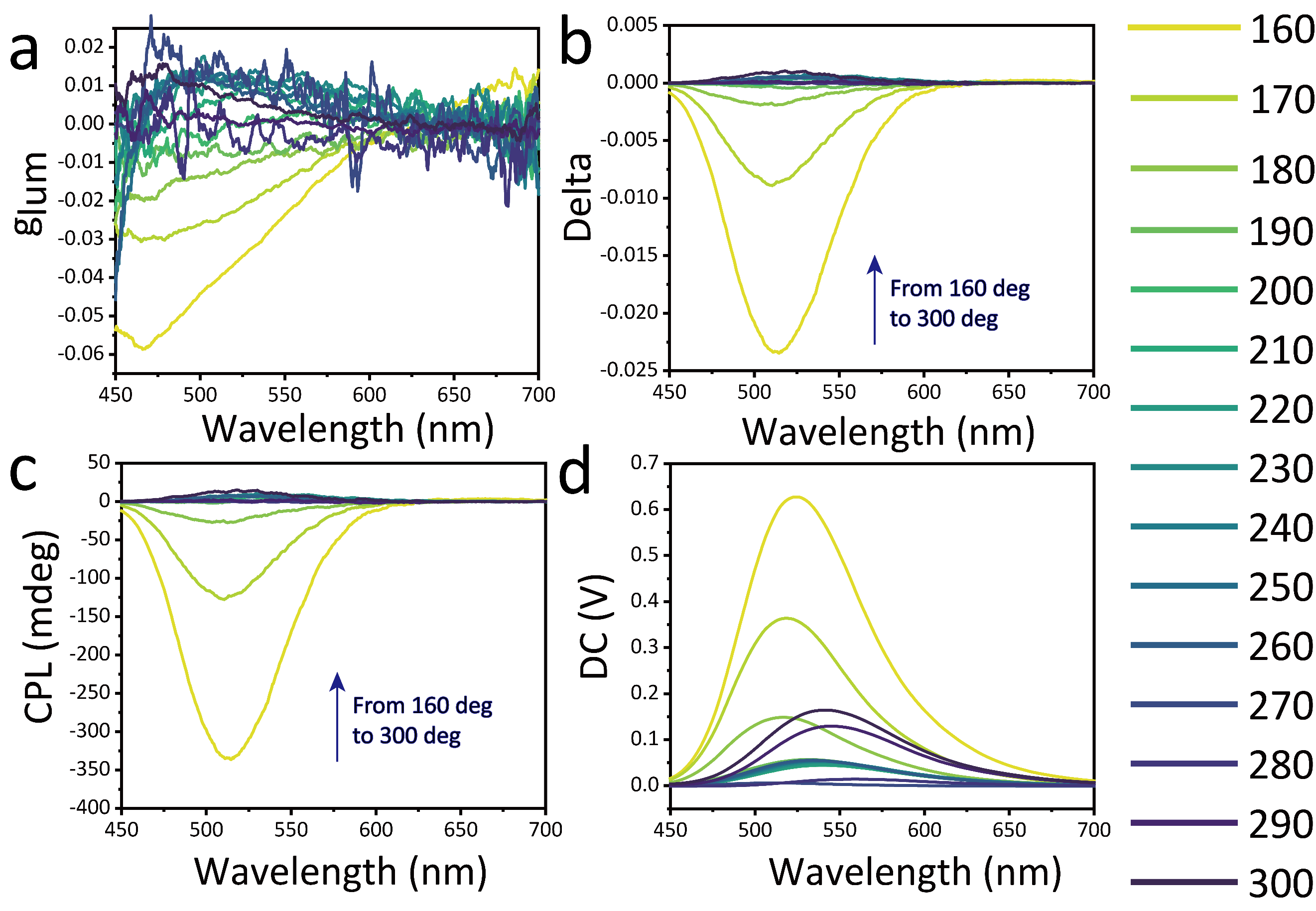


**Figure S7.** The direction of 142° in the figure was the angle of normal incidence of the excitation light onto the model benzil crystal face. In this case, the direction at 142° was parallel to the optical axis direction of the crystal. This figure shows the spectra of the change in CPL signal after changing the incidence angle of the excitation light (from 160° to 300°) when the wavelength of the incident excitation light was 360 nm. (a) the g_lum_ value spectra, (b) the delta value spectra, (c) the CPL spectra, and (d) the light intensity spectra.


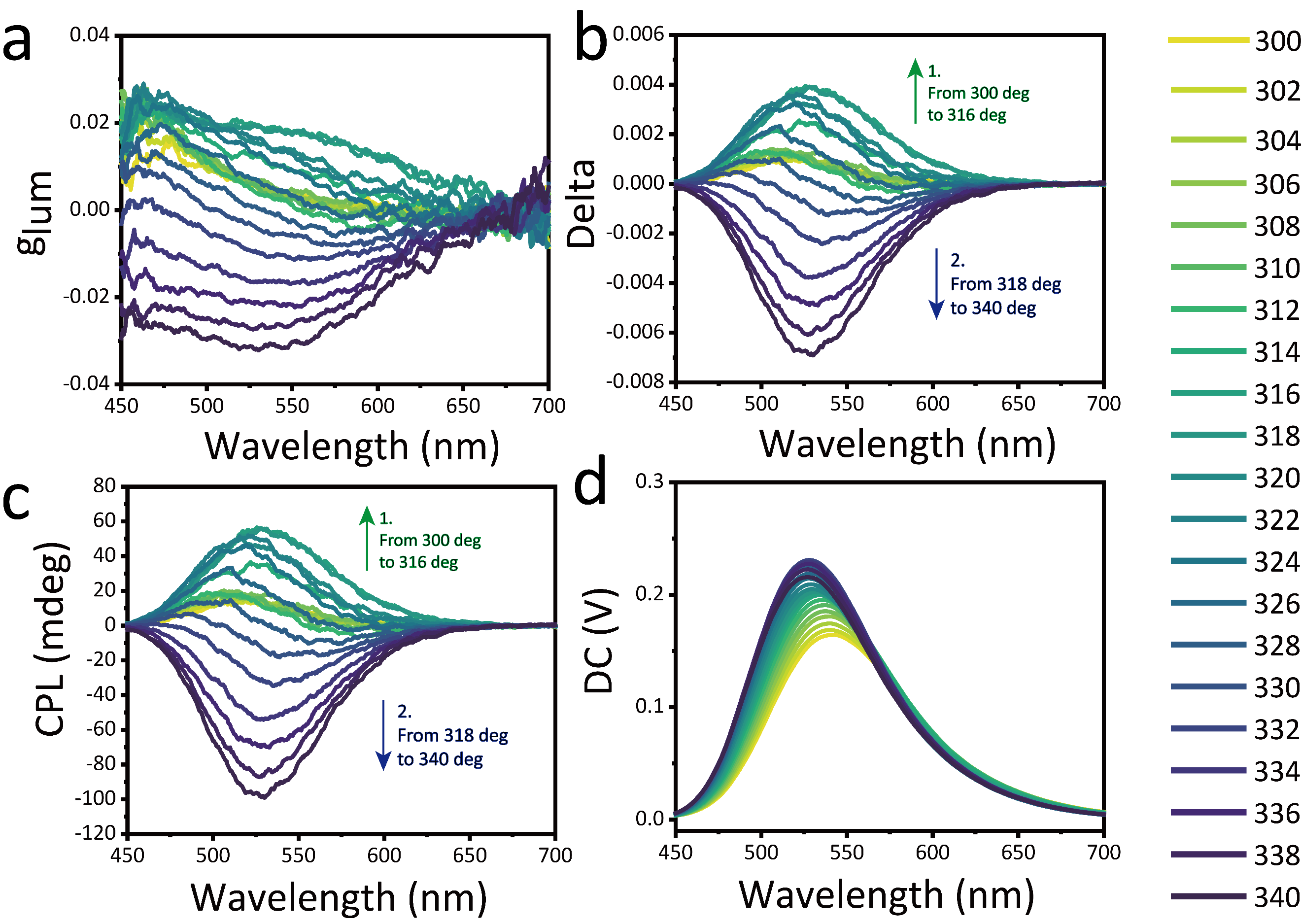


**Figure S8.** The direction of 142° in the figure was the angle of normal incidence of the excitation light onto the model benzil crystal face. In this case, the direction at 142° was parallel to the optical axis direction of the crystal. This figure shows the spectra of the change in CPL signal after changing the incidence angle of the excitation light (from 300° to 340°) when the wavelength of the incident excitation light was 360 nm. (a) the g_lum_ value spectra, (b) the delta value spectra, (c) the CPL spectra, and (d) the light intensity spectra.


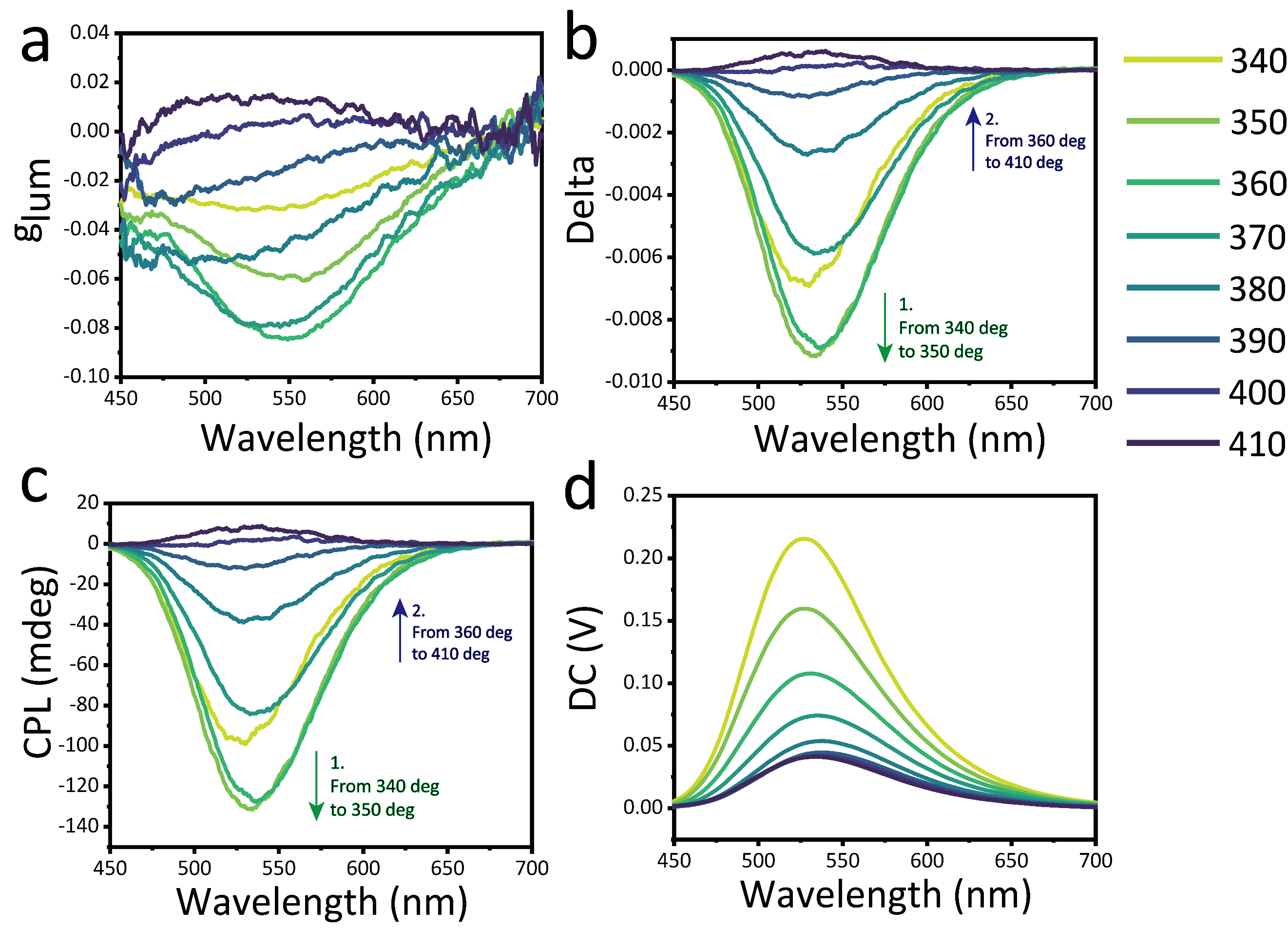


**Figure S9.** The direction of 142° in the figure was the angle of normal incidence of the excitation light onto the model benzil crystal face. In this case, the direction at 142° was parallel to the optical axis direction of the crystal. This figure shows the spectra of the change in CPL signal after changing the incidence angle of the excitation light (from 340° to 410°) when the wavelength of the incident excitation light was 360 nm. (a) the g_lum_ value spectra, (b) the delta value spectra, (c) the CPL spectra, and (d) the light intensity spectra.

## Angle-dependent experiments of the angle between the constant incident excitation light and the optical axis

The angle-dependent experiments of the angle between the constant incident excitation light and the optical axis. Here, the angle between the fixed incident angle and the optical axis of the crystal was 90 degrees. The wavelength of the incident excitation light was 360 nm, the incident direction of 142° was the normal incident angle of the crystal faces which the distance between the parallel faces is 5.110 mm, and the wavelength detected by the detector was 530 nm.


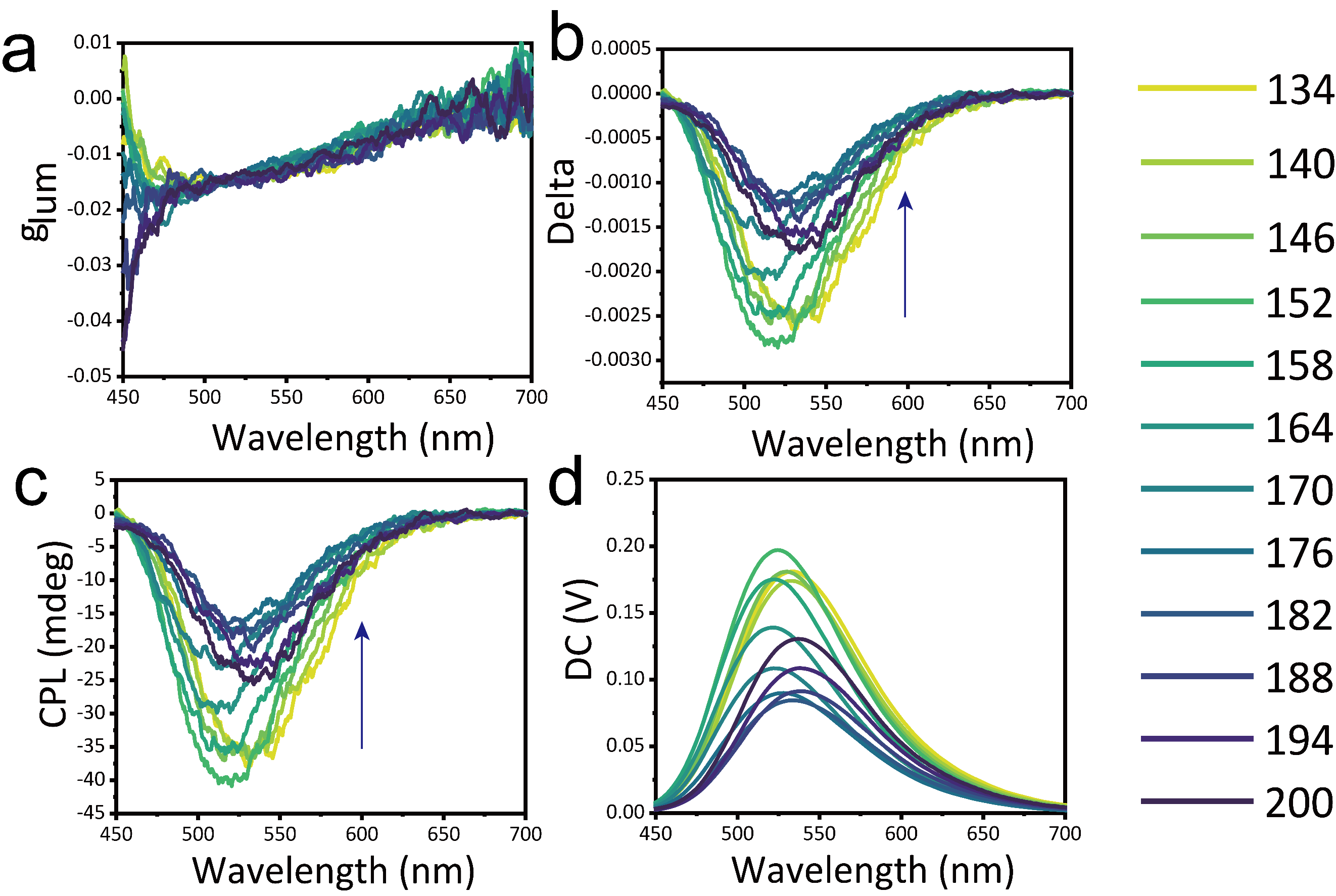


**Figure S10.** (a) the g_lum_ value spectra, (b) the delta value spectra, (c) the CPL spectra, and (d) the light intensity spectra of the CPL signal after changing the incident angle (from 134° to 200°) when the wavelength of the incident excitation light was 360 nm.


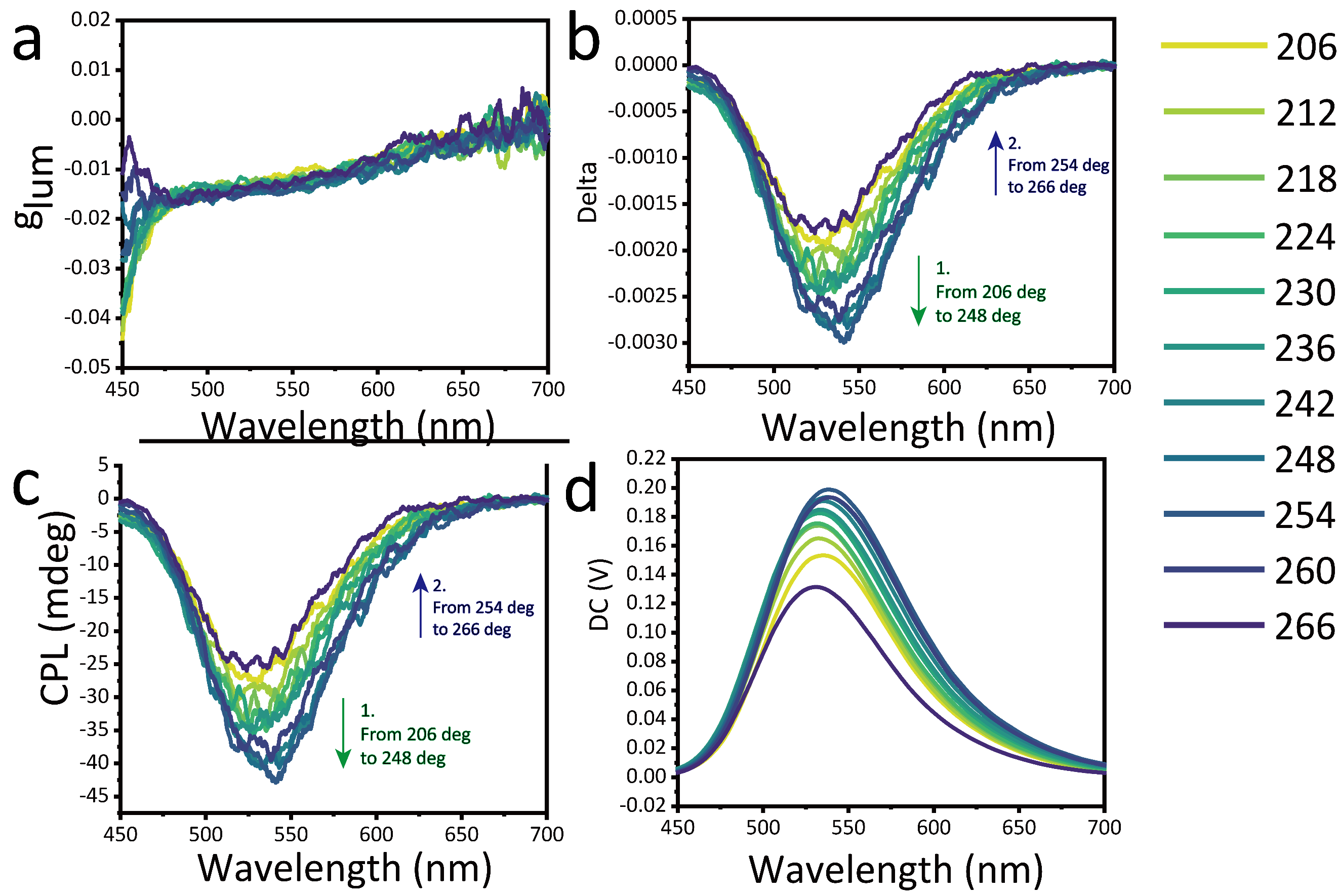


**Figure S11.** (a) the g_lum_ value spectra, (b) the delta value spectra, (c) the CPL spectra, and (d) the light intensity spectra of the CPL signal after changing the incident angle (from 206 to 266°) when the wavelength of the incident excitation light was 360 nm.


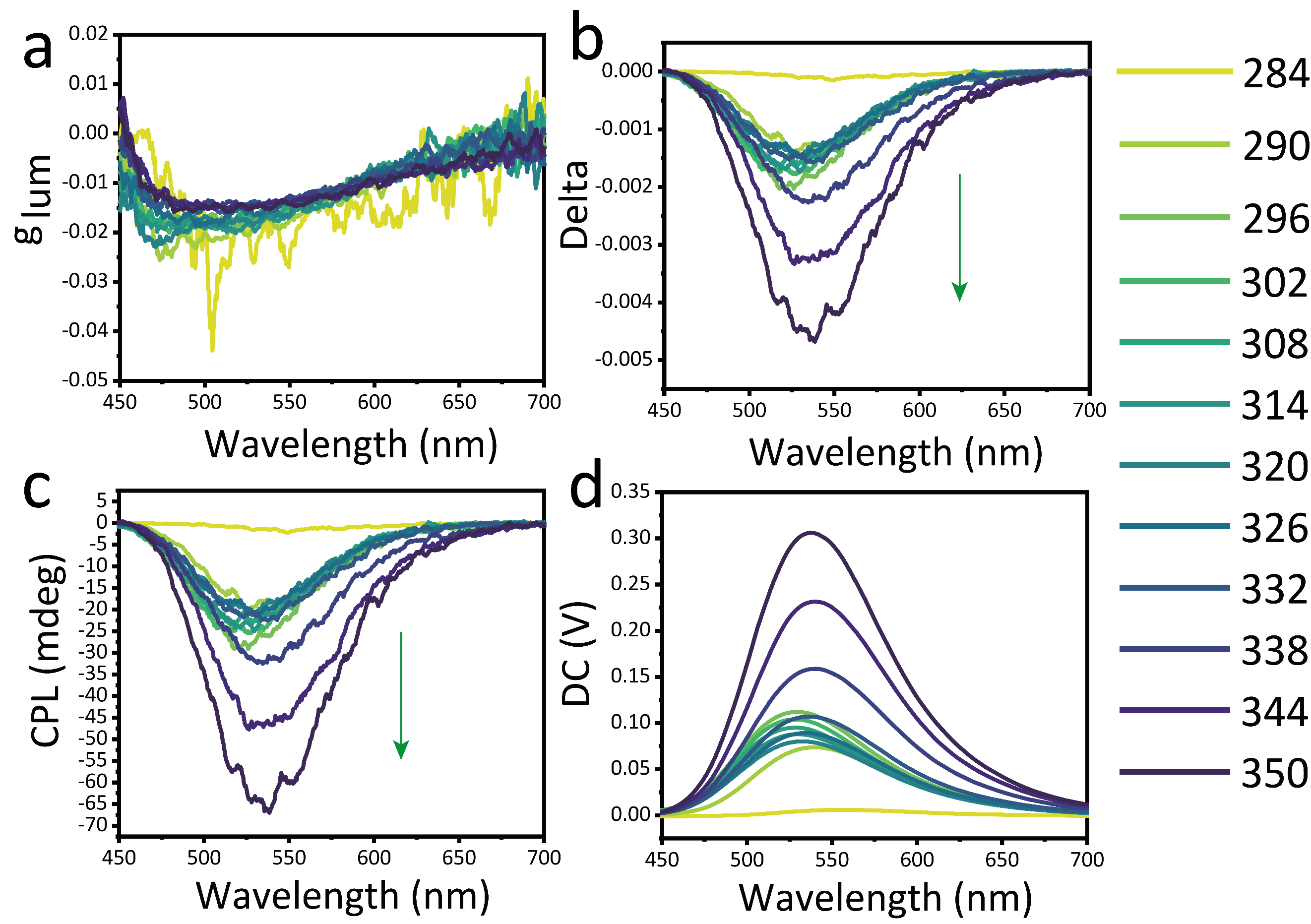


**Figure S12.** (a) the g_lum_ value spectra, (b) the delta value spectra, (c) the CPL spectra, and (d) the light intensity spectra of the CPL signal after changing the incident angle (from 284° to 350°) when the wavelength of the incident excitation light was 360 nm.


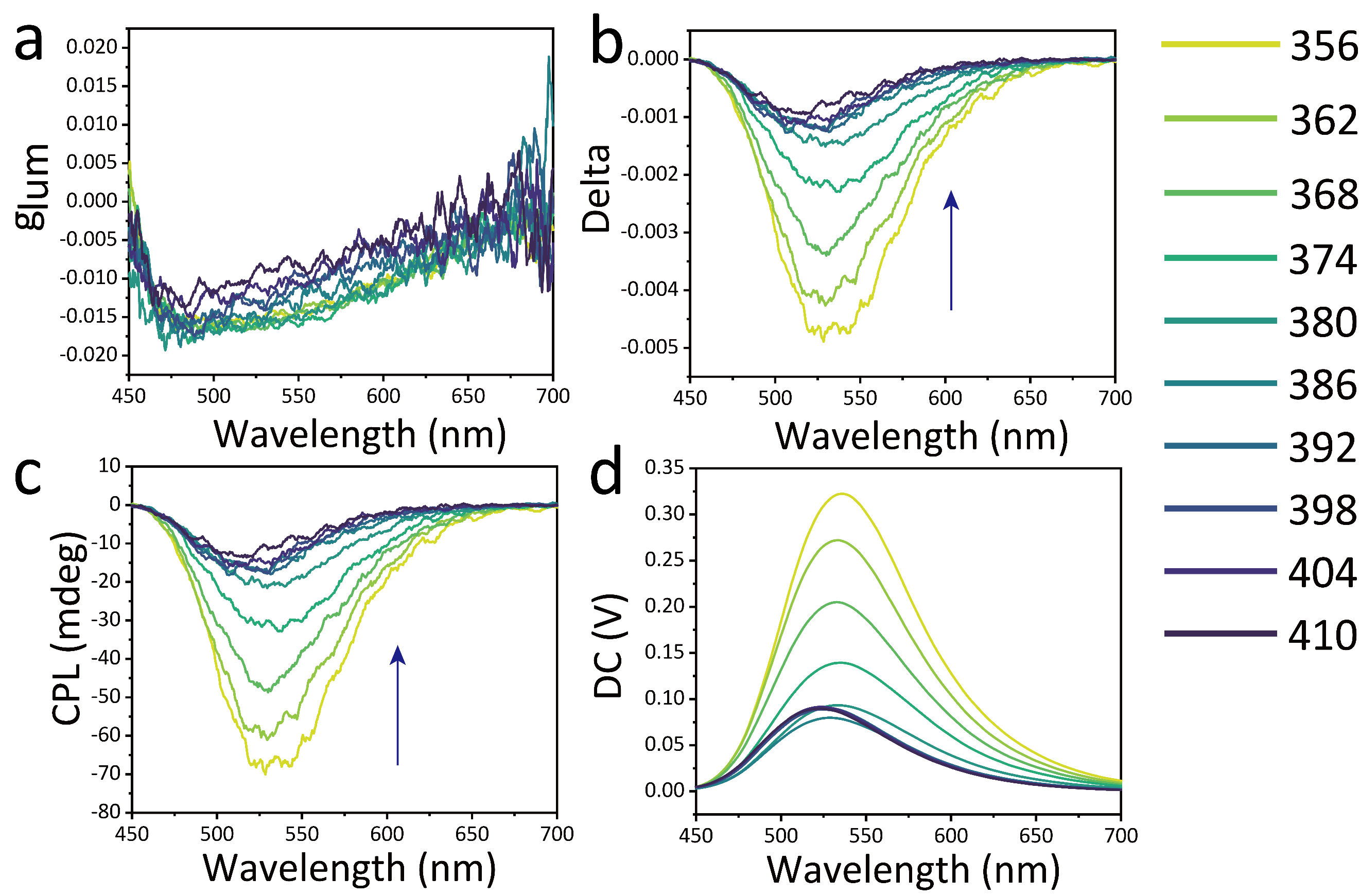


**Figure S13.** (a) the g_lum_ value spectra, (b) the delta value spectra, (c) the CPL spectra, and (d) the light intensity spectra of the CPL signal after changing the incident angle (from 356° to 410°) when the wavelength of the incident excitation light was 360 nm.

## Measurement of the entropy


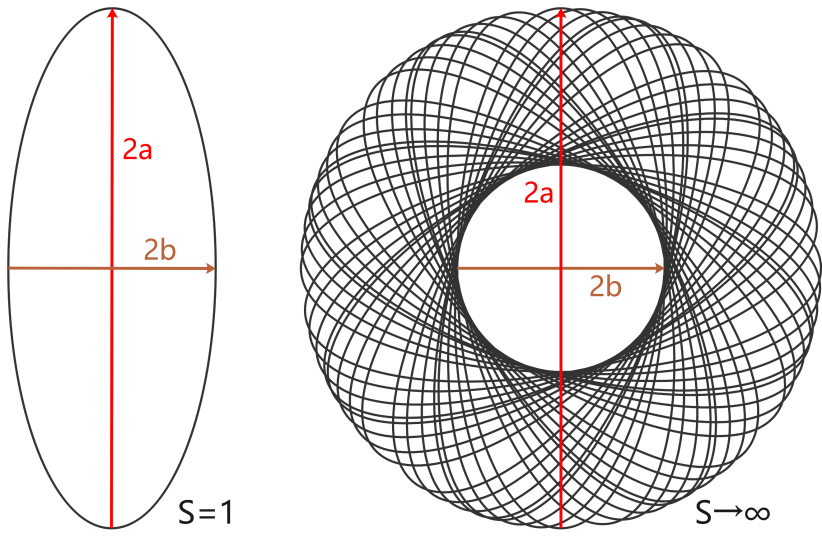


**Figure S14.** For the superposition state of many EPL, they represent anisotropic measurement character depending on the long axis and short axis of the corresponding ellipse. The isotropic measurement character could represent the condition when the measurement entropy is large.


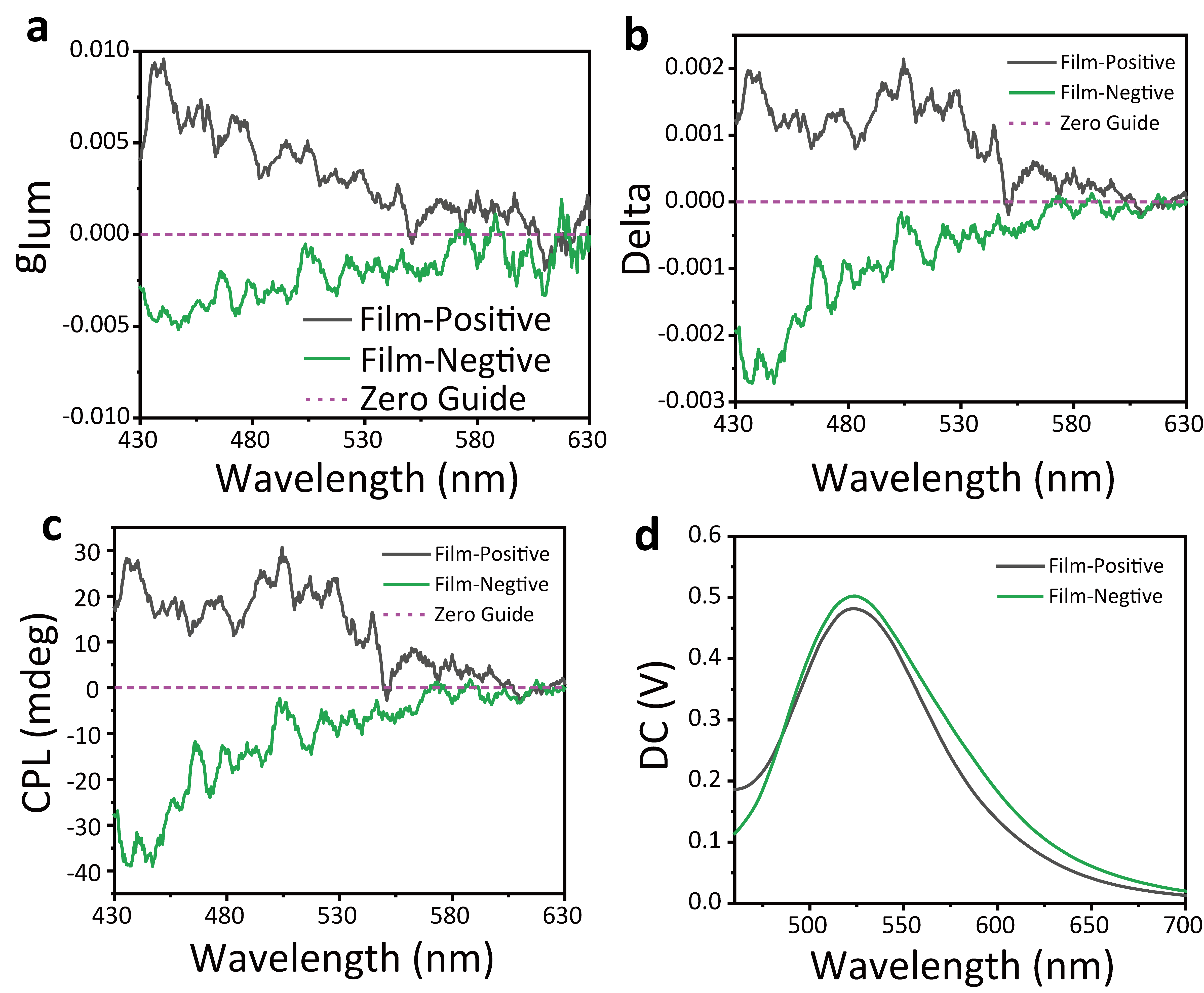


**Figure S15.** (a) the g_lum_ value spectra, (b) the delta value spectra, (c) the CPL spectra, and (d) the light intensity spectra of the CPL signal of benzil in films with opposite CPL states.

**Reference:**

1. Li, JJ, Wang, YA, Guo, W*, et al.* Large-scale synthesis of nearly monodisperse CdSe/CdS core/shell nanocrystals using air-stable reagents via successive ion layer adsorption and reaction. *J Am Chem Soc*. 2003; **125**(41): 12567-75.

2. Protasenko, V, Bacinello, D, Kuno, M. Experimental determination of the absorption cross-section and molar extinction coefficient of CdSe and CdTe nanowires. *J Phys Chem B*. 2006; **110**(50): 25322-31.

3. Qu, L, Peng, X. Control of photoluminescence properties of CdSe nanocrystals in growth. *J Am Chem Soc*. 2002; **124**(9): 2049-55.

4. Sun, Q, Wang, YA, Li, LS*, et al.* Bright, multicoloured light-emitting diodes based on quantum dots. *Nature Photon*. 2007; **1**(12): 717-22.

5. Pong, BK, Trout, BL, Lee, JY. Modified ligand-exchange for efficient solubilization of CdSe/ZnS quantum dots in water: a procedure guided by computational studies. *Langmuir*. 2008; **24**(10): 5270-6.

6. Landau, LD, Lifshit͡s, EM. *Electrodynamics of continuous media*. Oxford, New York,: Pergamon Press; 1960.

7. Yang, D-K, Wu, S-T. *Fundamentals of liquid crystal devices*: John Wiley & Sons; 2014.

8. Singh, UB, Dhar, R, Pandey, AS*, et al.* Electro-optical and dielectric properties of CdSe quantum dots and 6CHBT liquid crystals composites. *Aip Adv*. 2014; **4**(11): 117112.

9. Lehninger, AL, Nelson, DL, Cox, MM. *Lehninger principles of biochemistry*. New York: W.H. Freeman; 2013.

10. Yaghmaee, P, Karabey, OH, Bates, B*, et al.* Electrically Tuned Microwave Devices Using Liquid Crystal Technology. *Int J Antenn Propag*. 2013; **2013**: 824214.

11. Nichols, S. Coherence in polarimetry *Dissertation*. New York University; 2018.

12. Arteaga, O, Nichols, S, Kahr, B. Mueller matrices in fluorescence scattering. *Opt Lett*. 2012; **37**(14): 2835-7.

13. Zaffar, M. Fluorescence scattering in between excitation and emission as a depolarizing process: a Mueller matrix viewpoint. *J Opt Soc Am A*. 2022; **39**(7).

14. Naito, M, Iwahori, K, Miura, A*, et al.* Circularly polarized luminescent CdS quantum dots prepared in a protein nanocage. *Angew Chem Int Ed*. 2010; **49**(39): 7006-9.

15. Tohgha, U, Deol, KK, Porter, AG*, et al.* Ligand induced circular dichroism and circularly polarized luminescence in CdSe quantum dots. *ACS Nano*. 2013; **7**(12): 11094-102.

16. Okano, K, Taguchi, M, Fujiki, M*, et al.* Circularly polarized luminescence of rhodamine B in a supramolecular chiral medium formed by a vortex flow. *Angew Chem Int Ed*. 2011; **50**(52): 12474-7.

17. Rathore, R, Kumar, AS, Lindeman, SV*, et al.* Preparation and Structures of Crystalline Aromatic Cation-Radical Salts. Triethyloxonium Hexachloroantimonate as a Novel (One-Electron) Oxidant. *J Org Chem*. 1998; **63**(17): 5847-56.

18. Roitershtein, DM, Ziller, JW, Evans, WJ. Synthesis and Structure of a New Type of Sandwich-Like Yttrium Complex Derived from Tetraphenylethylene:  [Na(THF)6][Y(Ph2CCPh2)2]. *J Am Chem Soc*. 1998; **120**(44): 11342-6.

19. Guan, J, Wei, R, Prlj, A*, et al.* Direct Observation of Aggregation-Induced Emission Mechanism. *Angew Chem Int Ed* 2020; **59**(35): 14903-9.

20. Takaishi, K, Iwachido, K, Ema, T. Solvent-Induced Sign Inversion of Circularly Polarized Luminescence: Control of Excimer Chirality by Hydrogen Bonding. *J Am Chem Soc*. 2020; **142**(4): 1774-9.

21. Takaishi, K, Iwachido, K, Takehana, R*, et al.* Evolving Fluorophores into Circularly Polarized Luminophores with a Chiral Naphthalene Tetramer: Proposal of Excimer Chirality Rule for Circularly Polarized Luminescence. *J Am Chem Soc*. 2019; **141**(15): 6185-90.
